# Antihypertensive treatment in a general uncontrolled hypertensive population in Belgium and Luxembourg in primary care: Therapeutic inertia and treatment simplification. The SIMPLIFY study

**DOI:** 10.1371/journal.pone.0248471

**Published:** 2021-04-05

**Authors:** Tine De Backer, Bregt Van Nieuwenhuyse, Dirk De Bacquer

**Affiliations:** 1 Cardiovascular Center, University Hospital Ghent, Ghent, Belgium; 2 Medical Affairs Department Servier BeLux, Brussels, Belgium; 3 Department of Public Health and Primary Care, Ghent University, Ghent, Belgium; Shanghai Institute of Hypertension, CHINA

## Abstract

**Background:**

Despite effective treatments, blood pressure (BP) control remains suboptimal.

**Objective:**

The SIMPLIFY study aimed at identifying key factors related to therapeutic inertia in Belgium and Luxembourg, and evaluating how uncontrolled treated hypertension is managed in primary care.

**Methods:**

In a 2017 cross-sectional survey, 245 general practitioners (GP) collected routine clinical data from 1,852 consecutive uncontrolled (Office SBP/DBP ≥ 140/90 mmHg) hypertensive adult patients taking at least one antihypertensive drug.

**Results:**

Patients were 64 years old on average, 48% were women, 61% had dyslipidemia, 33% had diabetes mellitus and 22% had established cardiovascular disease. Half of the patients had 2 or more comorbidities. Patients had been treated for hypertension for an average period of 8 years, 40% of patients were in hypertensive stages 2–3, 44% were treated with monotherapy only, 28% with free combinations and 28% with at least one single pill combination (SPC). Therapeutic adherence was rated as ‘good’ in 62% of patients. AHT treatment was modified in 84% of patients.

In the group of patients with stage 2–3 hypertension, treatment remained unchanged in 5%. In the group of patients with stage 1 hypertension, treatment remained unchanged in 23% of patients. Patients treated for longer than 10 years were less likely to undergo treatment change (81%) compared to patients treated for less than 10 years (87%). Patients with 1 or 2 comorbidities were more likely to have their treatment modified (87%) compared to those with no comorbidities (61%) and those with ≥ 3 comorbidities (79%). If treatment was modified, a SPC was introduced in 90% of cases; 91% in stage 1–2 hypertension and 84% in stage 3 hypertension. SPCs were less frequently initiated in patients without comorbidities. Main reasons for the GPs to switch from a free association towards SPC were ‘better BP control’ (55%), ‘better therapeutic compliance’ (53%) and ‘simplicity for the patient’ (50%).

**Conclusion:**

The SIMPLIFY study confirms therapeutic inertia in hypertension management. After an average of 8 years hypertension treatment, almost 1 in 2 uncontrolled treated patients are on monotherapy. The key inertia drivers seem to be age, mild grade hypertension, isolated systolic hypertension, longer duration of antihypertensive treatment and better therapeutic adherence. When treatment is updated by the GP, the currently preferred strategy is switching towards SPC based therapy to improve BP control, and enhance therapeutic compliance by simplifying treatment for the patient.

**Trial registration:**

pharma.be visa number: VI 17/01/20/01

ISRCTN registered study: ISRCTN16199080.

## Introduction

Arterial hypertension is an important cause of death worldwide and one of the principal manageable risk factors for cardiovascular diseases [1]. Despite its profound impact on public health and the cost of health care, arterial hypertension remains largely underdiagnosed and undertreated. It is estimated that half of the patients with hypertension remain unaware of their disease, that the blood pressure (BP) of half of the treated hypertensive patients remains uncontrolled, and that half of the patients treated with antihypertensive drugs are non-adherent [2–5]. In a recent worldwide screening initiative during which 1,128,635 individuals had their blood pressure screened, up to 34.9% had hypertension. In this population worldwide unselected population, 20% received an antihypertensive treatment, but only 53.7% of these on-treatment patients had their blood pressure controlled [6]. General practitioners play a pivotal role in the early diagnosis and adequate treatment of patients with arterial hypertension.

On top of non-pharmacological measures to prevent and to treat arterial hypertension, the 2018 guidelines of the European Society of Hypertension and the European Society of Cardiology (ESC/ESH) [7] shifted the preferred treatment strategy from a step-based approach defined by treatment initiation with monotherapy followed by adding other antihypertensive drugs in case of uncontrolled hypertension, towards a single pill combination based strategy. Initiation of treatment with dual therapy based on an ACEi or ARB + calcium channel blocker or diuretic, preferably in a single pill, is advised in most patients, followed by the use of a single pill triple therapy of the aforementioned antihypertensive classes in case of uncontrolled on-treatment hypertension. the main reason for recommending the single pill combination strategy is to enhance patient adherence as well as reduce therapeutic inertia and hence improve blood pressure control [7].

Physician-related factors such as therapeutic inertia, that is, failure of modifying treatment regimens when abnormal clinical parameters are recorded, represent an important factor contributing to poor blood pressure control. Previous studies have shown that therapeutic inertia is largely the result of an overestimation of the blood pressure control by treating physicians. In uncontrolled hypertensive patients receiving at least 2 antihypertensive molecules, one out of 3 patients is not evaluated as having uncontrolled hypertension [8].

Studies on blood pressure control rates in Belgian hypertensive populations show control rates of 22% to 45% [8–12]. In Belgium, epidemiological data about the current strategies applied to handle uncontrolled on-treatment hypertensive patients in primary care are lacking. In order to provide real-world evidence on current strategies to cope with uncontrolled treated hypertension in usual primary care, we set up a large-scale cross-sectional survey (SIMPLIFY) with the aim to identify 1) the current preferred strategy in primary care in the contemporary treatment of hypertensive patients in Belgium and Luxemburg; 2) key factors associated with therapeutic inertia, and 3) the preferred strategy to improve BP control in uncontrolled on-treatment hypertensive patients. These results could help to raise awareness of therapeutic inertia and its, often unconscious, drivers so as to overcome these and eventually lead to better blood pressure control.

## Materials and methods

The observational cross-sectional SIMPLIFY survey used existing patient data recently registered in the patients’ medical record as part of routine clinical practice. Patients included in the study population were intended to be a representative sample of the Belgian and Luxembourgian adult uncontrolled on-treatment hypertensive population (≥ 18 years old, SBP ≥ 140 mmHg and/or DBP ≥ 90 mmHg) in primary practice. Patients with secondary hypertension were excluded from the study.

Over a period of 5 months, from June till November 2017, 270 GPs were recruited nationwide, with a well-balanced geographical distribution. In order to control for selection bias, the investigators were asked to collect the study data of the last 15 consecutive hypertensive patients seen in their clinical practice and corresponding to the inclusion criteria, so as to obtain a cross-sectional snapshot of the current situation in the study population, without information on prior and future management of these patients. At the time of consultation with the GP the patient provided oral informed consent to the GP to have data from their medical records used in research.

For each patient included in the study, a case report form (CRF) was provided to the GP in order to collect the following information from the patient medical record: age, gender, anthropometrics, presence of relevant comorbidities, office blood pressures as measured in routine clinical practice, antihypertensive drug use (international nonproprietary name INN), duration of antihypertensive treatment (years), other drugs relevant to the study, total number of drugs per day. These data were collected without formal definitions of the co-existing comorbidities, BP measurement or adherence status in order to truly reflect real current practice in 1^st^ line management of hypertension in Belgium and Luxembourg.

During the consultation the therapeutic decision to modify treatment, whereby modification could be any possible change to the existing treatment was noted, as well as which anti-hypertensive drugs were prescribed after the index consultation (used antihypertensive drugs (INN,), other drugs, total number of drugs per day)and the motivation for the GP to do so (therapeutic adherence, prognosis, cheaper, better BP control, simpler for the patient or other reason).

Besides these parameters the treating physician was also asked to report the estimated therapeutic adherence to the existing antihypertensive treatment. The treating physician was asked to give an estimate of the patient’s therapeutic adherence by choosing between “good, moderate, not adherent” during the index consultation.

Participating general practitioners were asked to measure blood pressure in the framework of this survey, hence representing real life data on BP measurements and interpretation in primary care. Hypertension was defined as a SBP of at least 140 mmHg and/or a DBP of at least 90 mmHg. Grade I hypertension was defined as having a SBP between 140–159 mmHg and/or a DBP between 90–99 mmHg, grade II hypertension was defined as having a SBP between 160–179 mmHg and/or a DBP between 100–109 mmHg, and grade III hypertension was defined as having a SBP of at least 180 mmHg and/or a DBP of at least 110 mmHg. Isolated systolic hypertension (ISHT) was defined as a SBP of at least 140 mmHg and a DBP of less than 90 mmHg, whereas isolated diastolic hypertension (IDHT) was defined as a SBP of less than 140 mmHg and a DBP of at least 90 mmHg. Therapeutic inertia was defined as no change in the antihypertensive treatment or management by the consulting physician despite an SBP and/or DBP ≥140 mmHg and/or ≥90 mmHg respectively during the index consultation.

The antihypertensive molecules used were subdivided in the following major classes: beta-blockers (BB), calcium channel blockers (CCB), ACE inhibitors (ACE-I-), angiotensin II receptor antagonists (ARB), thiazide-type diuretics, thiazide-like diuretics and “other diuretics”.

All study data collected were transferred by a clinical research associate into an electronic database. In order to ensure the quality of the data entry, a double-check was performed in a random sample of 5% of the entered records.

Patients’ demographics, risk factor profiles and use of medication were described according to means, standard deviations and proportions.

The study was conducted according to the quality standards for non-interventional studies outlined in the prevailing Code of Deontology of the Belgian pharmaceutical industry association (pharma.be).

## Results

### Study population

Overall, 245 GPs (78% of originally planned), participated and collected the study data. The GPs enrolled a total of 1852 patients on active antihypertensive treatment with uncontrolled blood pressure (SBP/DBP ≥ 140/90 mmHg).

The patient characteristics are shown in Table 1. Mean (SD) age was 64.1 (±13.0) years and 52% were men. The patients had a mean (SD) BMI of 27.6 (±4.7) kg/m^2^, with 70% of the patients being overweight and 26% being obese. Mean (SD) systolic blood pressure was 153.0 (±12.4) mmHg, and diastolic blood pressure was 88.5 (±9.4) mmHg. 40% of the patients were classified as having grade II (33%) or III (7%) hypertension. Prevalence of ISH was 41% whereas IDHT was observed in only 3% of the patients. The remaining 56% had both systolic and diastolic BP uncontrolled. The mean (SD) antihypertensive treatment duration before the index consultation was 8.4 (±6.3) years and treatment adherence was rated by the GP as “good” in only 62% of the patients. Almost all patients (96.6%) had at least one comorbidity, and up to 16% of the patients had a burden of at least 3 comorbidities. The most prevalent comorbidities were dyslipidaemia (61%) and diabetes (33%). In 22% of the patients a prior cardiovascular event had occurred.

**Table 1:** Baseline patient characteristics & prevalence of comorbidities in the study sample.

Baseline characteristics (n = 1852)	
	mean (SD) or %
Men n (%)	52%
Age (years)	64.1 (13.0)
BMI (kg/m2)	27.6 (4.7)
BMI ≥25 kg/m2	70%
BMI ≥30 kg/m2	26%
SBP	153.0 (12.4)
DBP	88.5 (9.4)
AHD treatment duration	8.4 (6.3)
Good adherence (%)	62%
Moderate adherence (%)	34%
Bad adherence (%)	4%
Grade 1 HT (%)	60%
Grade 2 HT (%)	33%
Grade 3 HT (%)	7%
ISHT (%)	41%
IDHT (%)	3%
S & D HT (%)	56%
Dyslipidaemia	61%
Diabetes	33%
Prior CV event	22%
Peripheral vascular disease	13%
Arrhythmias	13%
Renal Failure	8%
Heart Failure	5%
Other comorbidity	12%
None	3%
≥ 3 Comorbidities (%)	16%

Abbreviations: SD: Standard deviation; BMI: Body Mass Index; SBP: Systolic blood pressure; DBP: Diastolic blood pressure; AHD: Antihypertensive drug; HT: Hypertension; ISH: Isolated systolic hypertension; IDHT: Isolated diastolic hypertension; S & D HT: Systolic and diastolic hypertension.

### Preferred antihypertensive treatment

**Fig 1:**
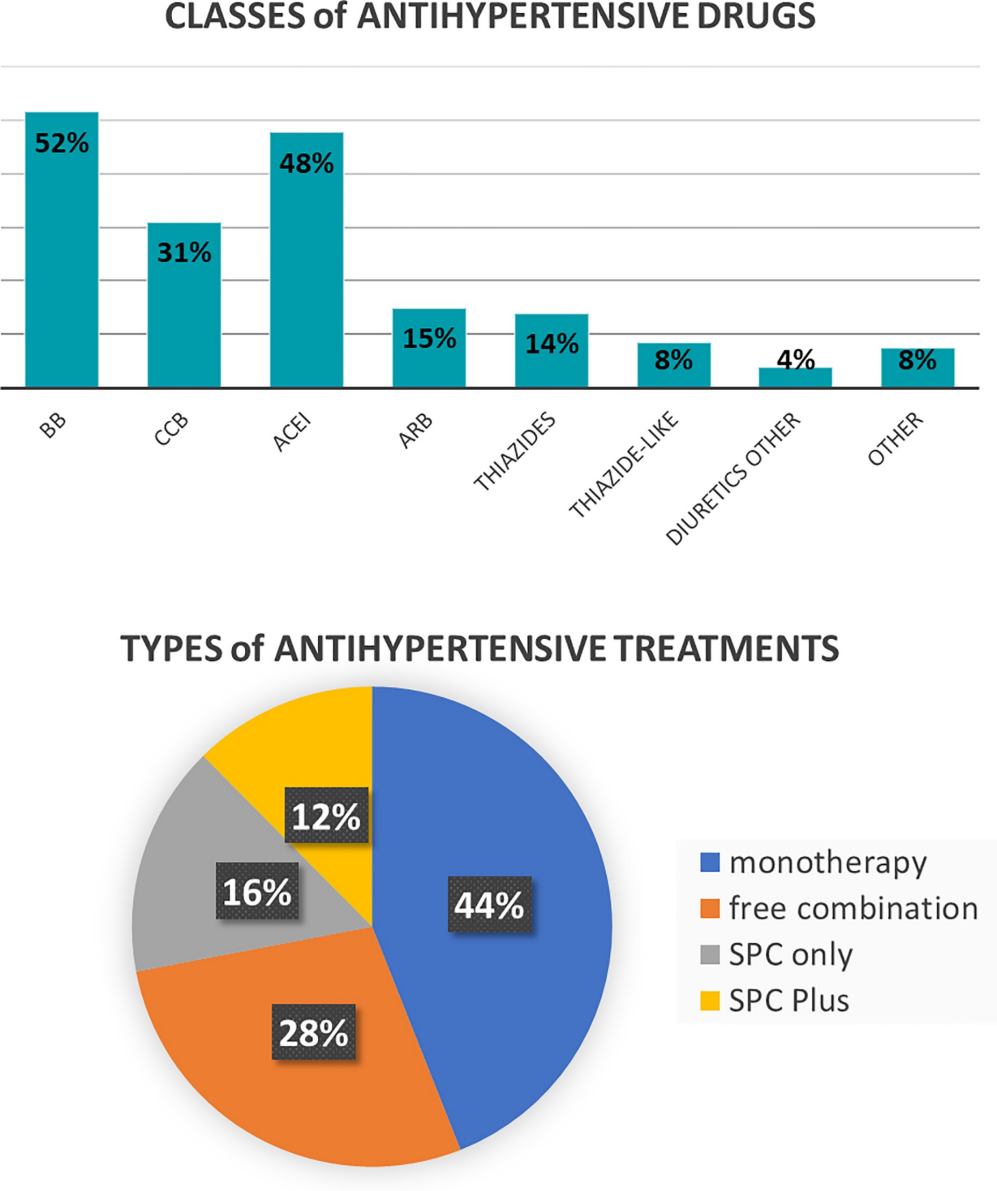
Classes and types of antihypertensive treatments used. Abbreviations: BB, beta-blocker; CCB, calcium channel blocker; ACEI, angiotensin converting enzyme inhibitor; ARB, angiotensin receptor blockers; SPC, single pill combination. SPC only = exclusive use of SPCs/SPC Plus = use of SPCs and single molecule antihypertensive drugs.

Beta-blockers and ACEI were used in 52% and 48% of the patients, followed by CCB in 31%, ARB in 15% and thiazide diuretics in 14%. Monotherapy was used in 44%, free drug combinations in 28% and “SPC only” (i.e. no use of a free combination of individual pills) and SPC plus (i.e. combined use of SPCs and individual pills) in respectively 16% and 12% (Fig 1). Beta-blockers and ACEIs were used in 1 out of 3 patients who were in monotherapy, whereas ARBs and calcium channel blockers monotherapy in 1 out of 5 patients and diuretics monotherapy in 1 out of 10 patients.

### Key characteristics related to therapeutic inertia

**Fig 2:**
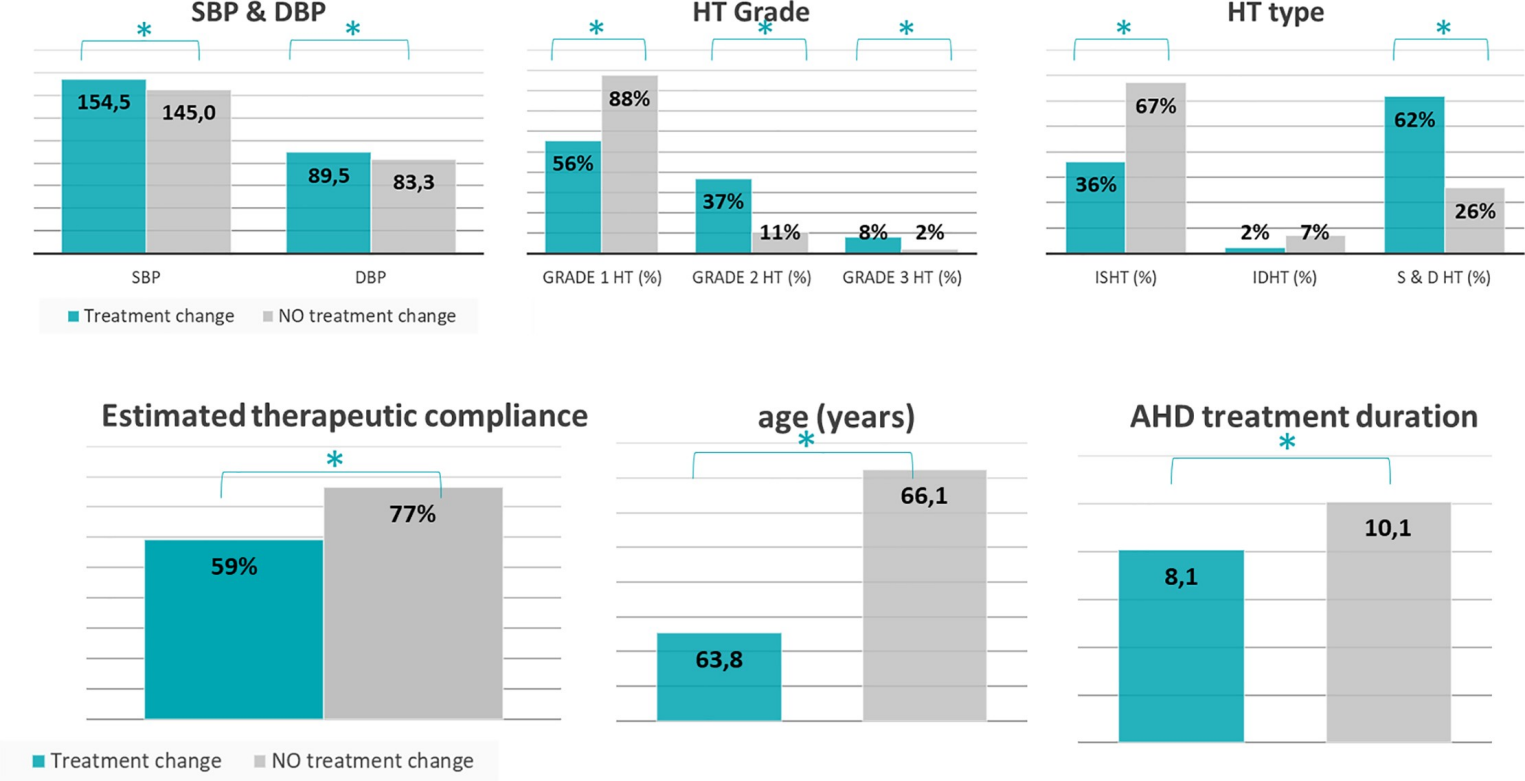
Patient characteristics in relation to to therapeutic inertia. Abbreviations: AHD: Antihypertensive drug, SBP: Systolic blood pressure; DBP: Diastolic blood pressure; HT: Hypertension; ISHT: Isolated systolic hypertension; IDHT: Isolated diastolic hypertension.

Antihypertensive treatment was not modified in 16% of the uncontrolled on-treatment hypertensive patients. This therapeutic inertia seems associated with older age (66.1 vs 63.8 years old), a longer history of prior antihypertensive treatment (10.1 vs 8.1 years of antihypertensive treatment), and a higher rate of good therapeutic adherence (77% vs 59%) (Fig 2). Furthermore therapeutic inertia seems associated with a lower mean SBP and DBP (145.0 mmHg vs 154.5 mmHg), with grade I hypertension (88% vs 56%) and with isolated systolic hypertension (67% vs 36%). Also, there seems a trend that GPs are less likely to change treatment (22% vs 15%) in patients with 2 or more co-morbidities.

### Preferred treatment strategy to improve BP control

In the uncontrolled on-treatment patients whose treatment was modified, the preferred type of treatment in order to improve BP control was in all cases (i.e. regardless of the previous treatment type) the use of SPCs, with 86%, 72%, 83% and 59% switching from monotherapy, free combination, SPC only, or SPC plus respectively towards SPC only. The 83% from SPC only towards SPC only represents a switch in dosage or in classes comprising the SPC.

The preferred classes of antihypertensive drugs to improve BP control were ACEIs, and BBs and to a lesser extent calcium channel blockers, as shown in Table 2.

**Table 2:** Preferred switch when no inertia occurs (a) towards treatment class and (b) towards treatment type.

**a. Preferred switch towards angiotensine converting enzyme inhibitors and beta-blockers**									
Preferred strategy treatment class									
		N	After switch						
			BB	CCB	ACEi	ARB	Thiazides-type	Thiazide-like	Other Diuretics
Before switch	BB	807	90%	19%	89%	5%	8%	9%	2%
	CCB	459	40%	72%	85%	11%	8%	27%	3%
	ACEi	773	53%	42%	97%	2%	3%	26%	2%
	ARB	188	53%	44%	45%	54%	36%	13%	4%
	Thiazides-type	183	70%	35%	78%	16%	28%	20%	1%
	Thiazide-like	122	36%	54%	93%	5%	4%	68%	2%
	Diuretics other	57	63%	25%	81%	7%	5%	23%	37%

**b. Preferred switch towards SPC**					
Preferred strategy to switch treatment type					
	After switch				
		monotherapy	free combination	SPC only	SPC Plus
Before switch	monotherapy	8%	3%	86%	2%
	free combination	2%	6%	72%	21%
	SPC only	3%	0%	83%	14%
	SPC Plus	3%	1%	58%	38%

Abbreviations: BB: beta-blocker; CCB: calcium channel blocker; ACEI: angiotensine converting enzyme inhibitor; ARB: angiotensin receptor blockers; SPC: single pill combination. SPC only = exclusive use of SPCs / SPC Plus = use of SPCs and single molecule antihypertensive drugs.

### Key drivers to modify treatment

**Fig 3:**
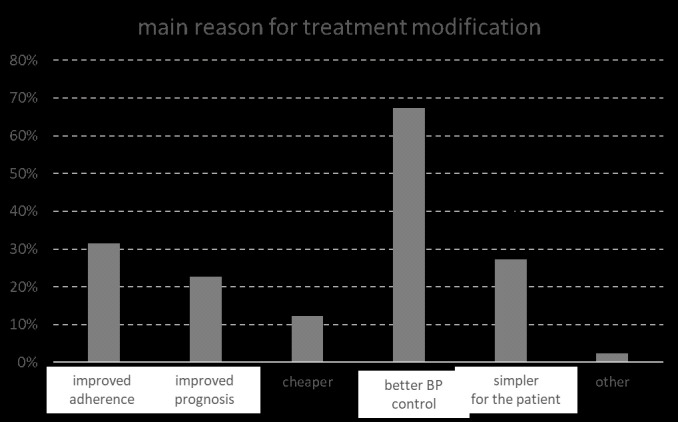
Reasons indicated by the GP for modifying antihypertensive treatment.

In 84% of the uncontrolled hypertensive patients antihypertensive treatment was modified. The main reasons for general practitioners to modify the treatment of uncontrolled on-treatment hypertensive patients were multiple, but were mainly better BP control (67%), followed by improved therapeutic adherence (31%) and simpler use for the patient (27%) (Fig 3).

## Discussion

In this cross-sectional survey in a high-risk population of 1852 uncontrolled treated hypertensive patients with a long treatment duration (8.4 years) it was observed that in 16% of the cases treatment was left unchanged, indicating a sub-optimal management of these high-risk patients. This proportion of observed therapeutic inertia is likely to be biased and is likely to be an underestimation of the true situation in clinical practice given the acknowledgment of study protocol and object are likely to subconsciously change the clinical decision of the participated GPs on whether the drug prescription should be modified.

There was a preferred use of beta-blockers (52%) and ACEIs (48%) followed by CCBs (31%). The relatively large use of beta-blockers is in line with the overall high risk profile of our patients with substantial comorbidity burden. Antihypertensive treatments were given as monotherapy in almost half of all patients, followed by free combinations in one third and SPC only or SPC plus in almost another third of patients. In 84% of the patients in whom treatment was modified, beta-blockers and ACEIs were still the preferred classes, with a preferential switch to mainly SPC type treatments. The switch towards a situation with SPC treatments in 90% of the patients may be an overestimation of the true situation in clinical practice. The acknowledgment of study protocol and object are likely to subconsciously change the clinical decision of the participated GPs on whether SPCs should be prescribed.

Uncontrolled blood pressure, excluding true resistant hypertension, is an issue of the patient, physician and the community.

As emphasized in the ESH/ESC 2018 hypertension guidelines, low adherence to treatment is a major cause of poor BP control and should hence be monitored.

Thirty-eight percent of the patients in our study were judged by the GP to have insufficient treatment adherence.

Besides patient non-adherence, longstanding therapeutic inertia by the physician is one of the key contributors to insufficient blood pressure control. Reasons for this therapeutic inertia are multiple. In our study, therapeutic inertia seemed associated with age, hypertension stage, isolated systolic hypertension, longer treatment duration and better therapeutic adherence.

Therapeutic inertia, defined as failure of healthcare providers to initiate or intensify therapy when indicated [13], might be an obstacle to good management of hypertension, which involves recognition of the abnormality and initiating and/or intensifying treatment until therapeutic goals are reached.

Therapeutic inertia is a major cause of not reaching target blood pressure in regular clinical practice. Investigator inertia has even been observed in clinical study settings. Kjeldsen et al analysed the LIFE and ASCOT data to find out why SBP levelled off after 6 months of blinded treatment with many patients with SBP > 140 mmHg remaining at their initial dose and not being uptitrated. Their conclusion was that there was no other reason than investigator inertia for the levelling off of the BP [14].

In our study, the fact that many patients remained on monotherapy, despite their BP being above target, points towards therapeutic inertia. The underlying causes of this therapeutic inertia are unclear. While it was actively asked why the GP did modify the treatment, it was not actively asked why the GP did not modify the treatment, which is certainly a complex entity. However, we could identify factors associated with therapeutic inertia: older age, grade 1 hypertension, isolated systolic hypertension, longer treatment duration and better therapeutic adherence.

Gil-Guillén et al, quantified diagnostic and therapeutic inertia in hypertension and identified patient-associated variables in a cross-sectional, multicentre study in 35000 people in 428 health centres and primary care centres, and found that diagnostic inertia and therapeutic inertia were present in respectively 32.5% and 37% of the cases [15]. Factors associated with both diagnostic and therapeutic inertia were isolated systolic or diastolic high BP, a finding similar with the observation in our study regarding therapeutic inertia. Factors associated with therapeutic inertia were DM type 2, CHD, stroke and BP values, similarly to our survey where the presence of co-morbidities and grade 1 hypertension were associated with therapeutic inertia.

The “Objetivo Kontrol” study [16] analysed therapeutic inertia in primary care and in specialist care with the primary objective of constructing a validated questionnaire to detect therapeutic inertia in hypertension. Redon et al aimed to identify the factors associated with therapeutic inertia in the management of arterial hypertension during consultation and to develop a predictive model to estimate the probability of inertia. Therapeutic inertia occurred in 78% of patients in primary care and in 59% in hospital care. Risk factors for therapeutic inertia were treatment in primary care, male sex, older age, BP values close to normal, use of more than one antihypertensive drug, treatment with an ARB and more than 6 physician visits for hypertension control a year. The predictive model was internally and externally valid and explained 25% of the variation in therapeutic inertia. This means that still other factors play a role in therapeutic inertia, highlighting its complexity. Involving the patient and motivating the physician with regard to hypertension management might help in improving blood pressure control [17].

To address inertia, adequate interventions are needed. Kjeldsen and the VALUE investigators [14] launched the systolic BP initiative to improve BP control rates via educational and organizational measures. All patients with a SBP 10 mmHg or more above target were listed up and the leading physicians were mailed. An increase in BP control was seen that stabilized at about 6 months. The ACCOMPLISH investigators showed that control rates were much higher with initial combination therapy with around 80% of patients having BP <140/90 mmHg at 30 months.

In our study, the GPs who modified the treatment, which consisted mainly of a switch towards single-pill combination, indicated better BP control, better therapeutic adherence and a simpler treatment regimen as the main drivers to intensify therapy. In the ACHIEVE study, a cross-sectional observational survey in primary care in Belgium and Luxembourg, Leeman et al. evaluated the actual blood pressure control rate and its estimation by general practitioners, the use of single-pill or free combinations, and the attitude towards single-pill combinations in primary care [8]. In patients requiring at least two antihypertensive drugs, blood pressure control rate remained low and was overestimated by general practitioners. Free combinations remained largely used although many general practitioners were willing to shift to single-pill combinations. Studies have shown that patients who immediately started a combination of antihypertensive medicines, as is recommended in the current ESC hypertension guidelines [7], reached the desired blood pressure faster than a control group who first started monotherapy and later titrated or added medication. In the latter group, after three years, only one in three patients was still receiving combination therapy, compared to almost 80% of the group that had received combination therapy from the start. This illustrates how early combination therapy can help prevent therapeutic inertia. And with important results: during the three years of follow-up, patients who had started combination therapy immediately had a 20% lower mortality rate and a 16% lower risk of cardiovascular diseases [18].

In addition, combinations of therapies can reduce side effects, for example by adding a thiazide-type diuretic to a calcium-antagonist, the symptoms of peripheral edemas can be reduced [19].

Finally, treatment simplification could improve adherence and could represent a tool to decrease therapeutic inertia in order to improve blood pressure control rate, which has been shown to lead to reduced morbidity and mortality [20].

This study has several limitations. Physicians participated to the study on a voluntary basis, which may reflect a particular interest in the management of high blood pressure, and therefore maybe not fully representative for the overall GP population. Also the patients may not fully reflect the overall general population of hypertensive patients in Belgium and Luxembourg. The data were collected without formal definitions of the co-existing comorbidities, BP measurement or adherence status-which were left to the GPs judgement- in order to truly reflect current practice in 1^st^ line management of hypertension in Belgium and Luxembourg. The study is a pure cross-sectional design study, so no predictions or causal relationships can be made. The strengths of the study might be the simplicity of the study protocol (which might be seen as a weakness at the same time). Although there might be some selection bias, the study reflects well the common current management of hypertension in GP practices.

## Conclusion

The SIMPLIFY study confirms the existing therapeutic inertia in treatment of arterial hypertension in routine clinical practice conditions in Belgium and in Luxembourg. After an average of 8 years hypertension treatment, almost 1 in 2 uncontrolled treated patients are still on monotherapy. The key inertia drivers seem to be age, mild grade hypertension, isolated systolic hypertension, longer duration of antihypertensive treatment and better therapeutic adherence. When treatment is updated by the GP, the currently preferred strategy is switching towards SPC-based therapy to improve BP control, and enhance therapeutic adherence by simplifying treatment for the patient.

## Supporting information

S1 Checklist TREND statement checklist. (PDF)

S1 File (DOC)

S2 File (DOC)

S3 File (PPTX)

S4 File (DOC)

S5 File (DOC)

S6 File (DOC)
