# Supplementary figures and images for: Antihypertensive treatment in a general uncontrolled hypertensive population in Belgium and Luxembourg in primary care: Therapeutic inertia and treatment simplification. The SIMPLIFY study
Source: PLoS One. 2021 Apr 5;16(4):e0248471. doi: 10.1371/journal.pone.0248471 (PMC8021160; doi:10.1371/journal.pone.0248471)

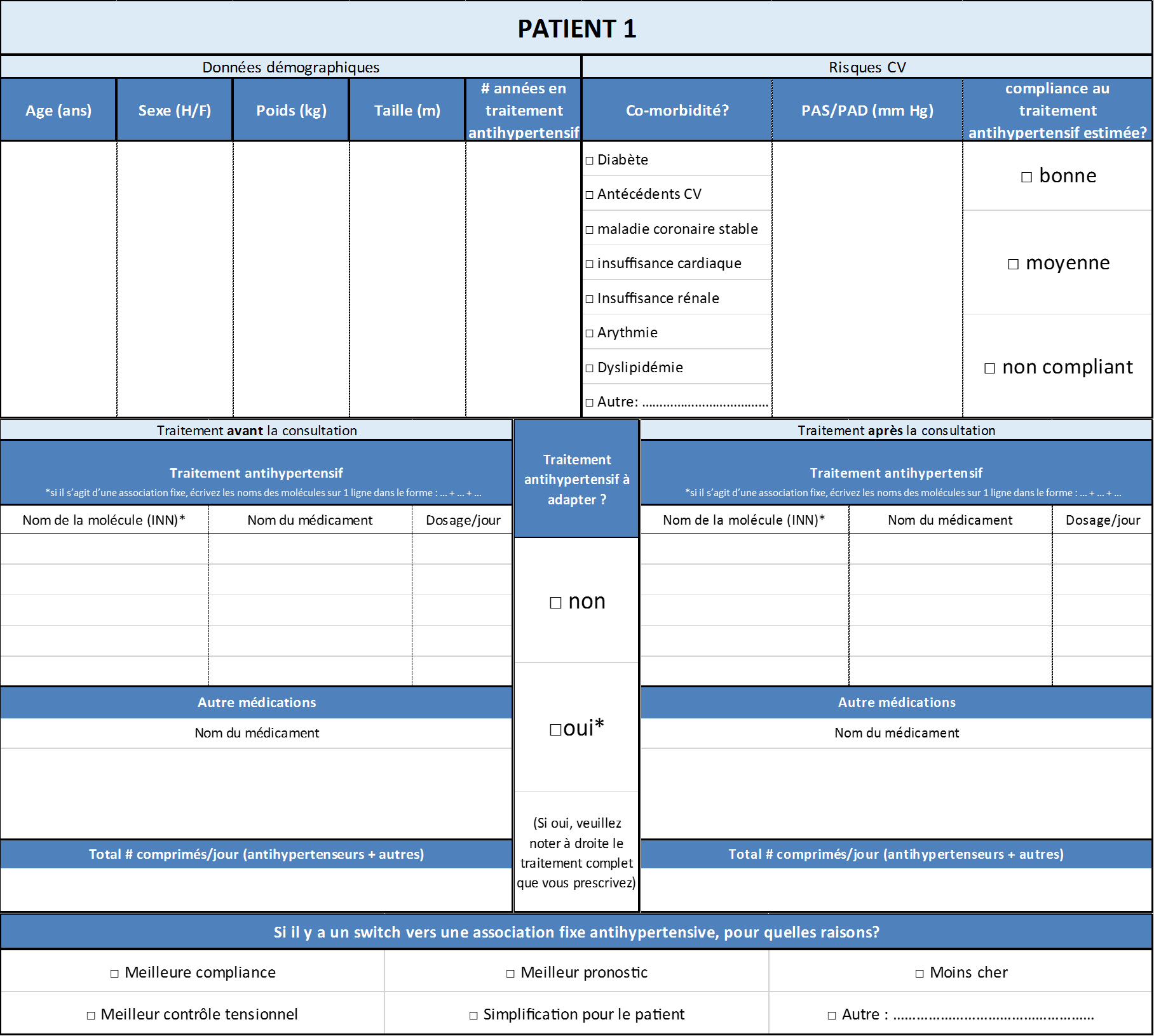

Supplement: S1 File — (DOC) [file pone.0248471.s002.doc]

## Slide 1
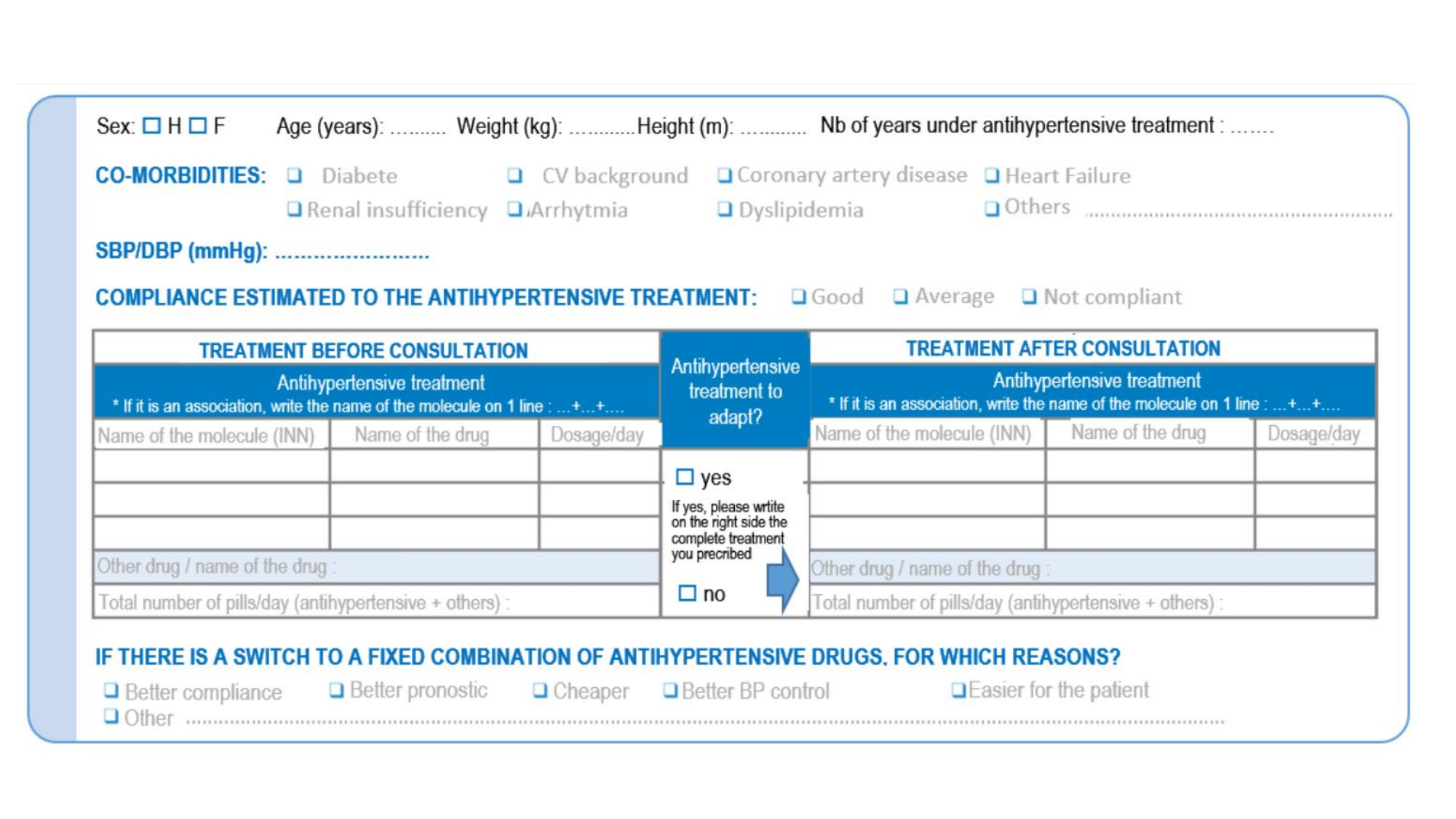

#

Supplement: S3 File — (PPTX) [file pone.0248471.s004.pptx]
