# Supplementary material for: Antihypertensive treatment in a general uncontrolled hypertensive population in Belgium and Luxembourg in primary care: Therapeutic inertia and treatment simplification. The SIMPLIFY study
Source: PLoS One. 2021 Apr 5;16(4):e0248471. doi: 10.1371/journal.pone.0248471 (PMC8021160; doi:10.1371/journal.pone.0248471)

**SIMPLIFY**

**Cross-sectional survey evaluating the motivation and key drivers of general practitioners to SIMPLIFY or intensify antihypertensive treatment in the Belgian general uncontrolled hypertensive population treated with at least one anti-hypertensive agent**

Naam van de onderzoeksarts : ............................................................................ Stempel

Adres : .................................................................................................................

............................................................................................................................................

Handtekening :

Visa Pharma.be : N° verkregen op .../.../...

Contactpersoon Servier : ...........................................................


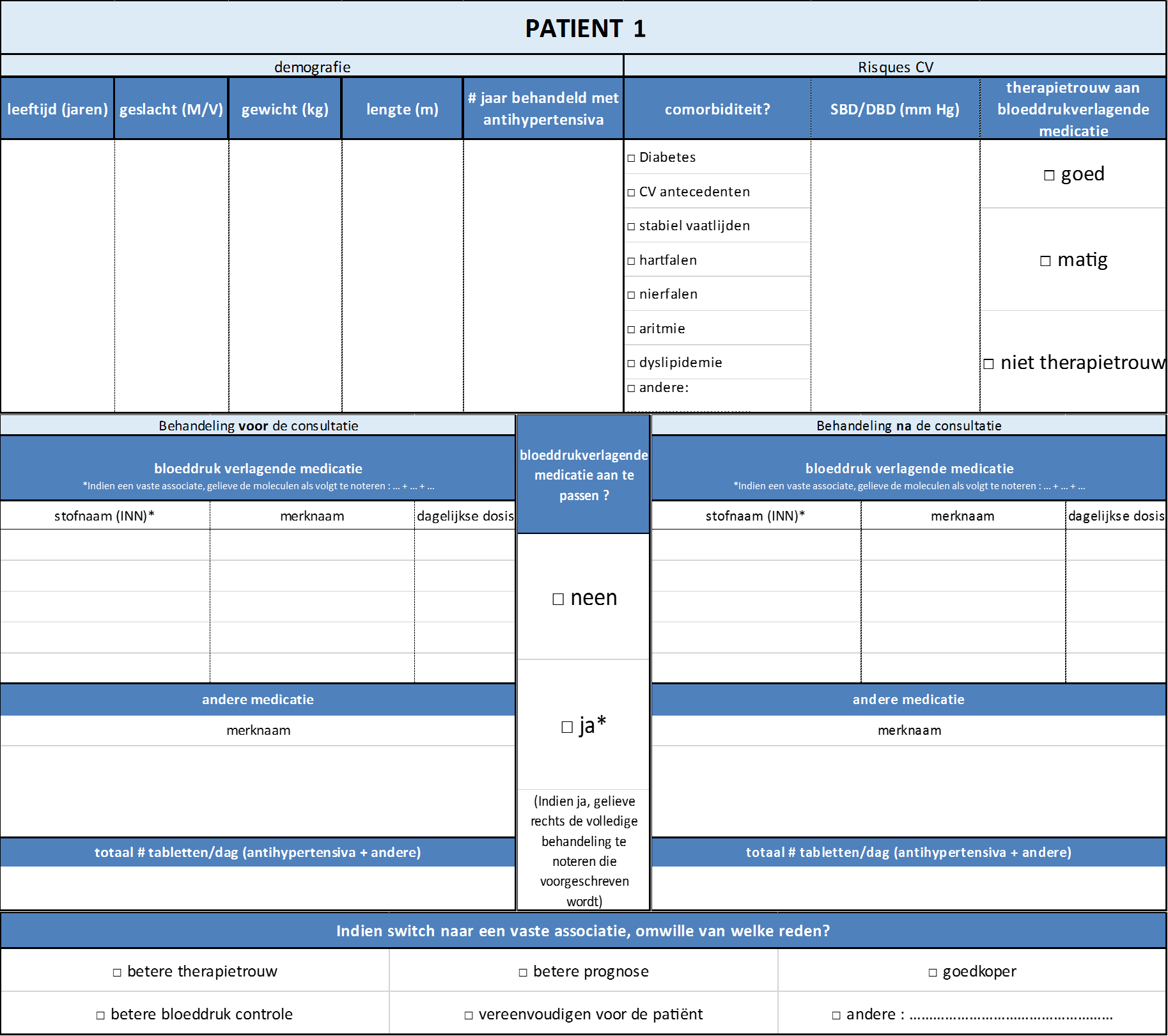


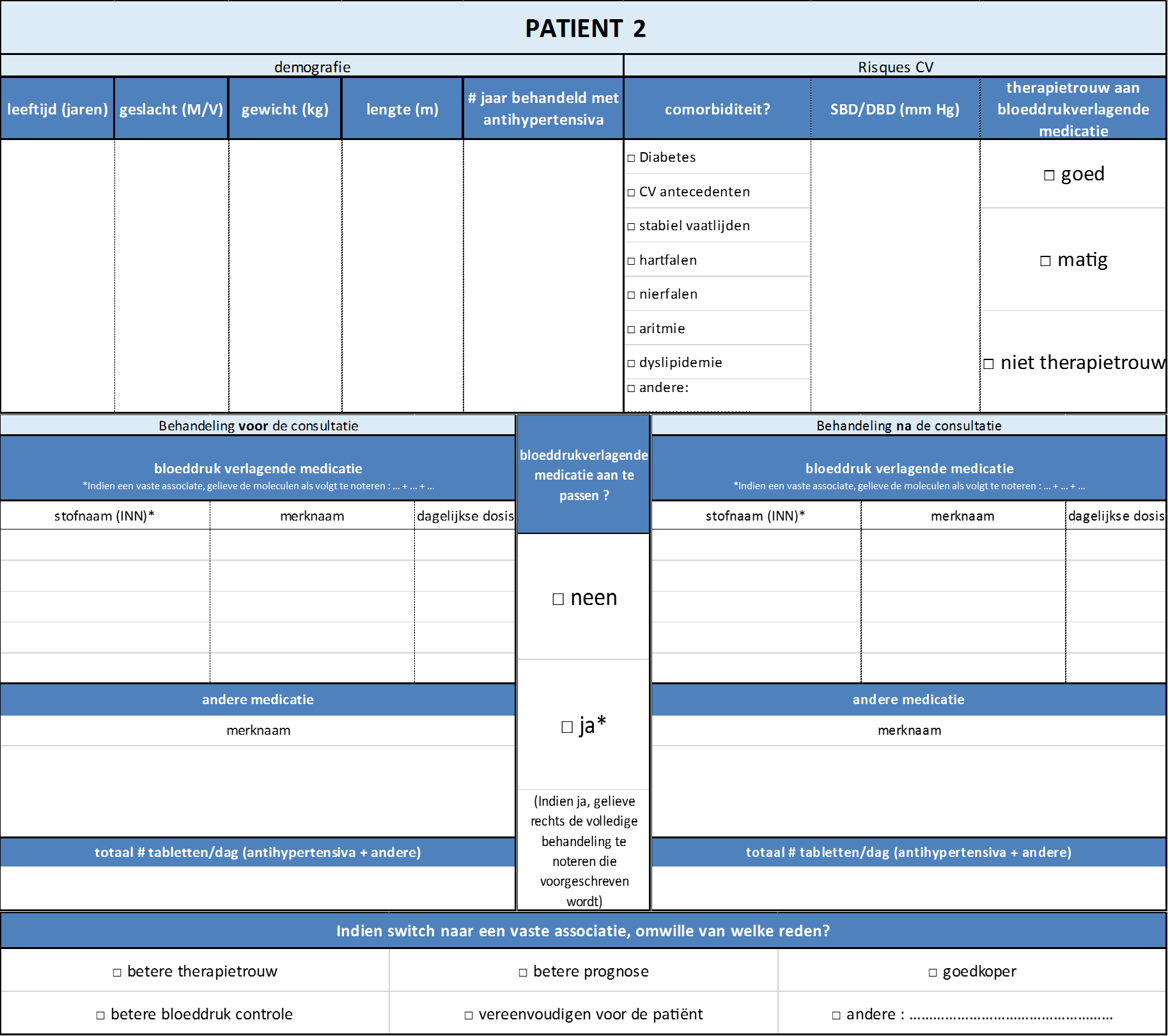


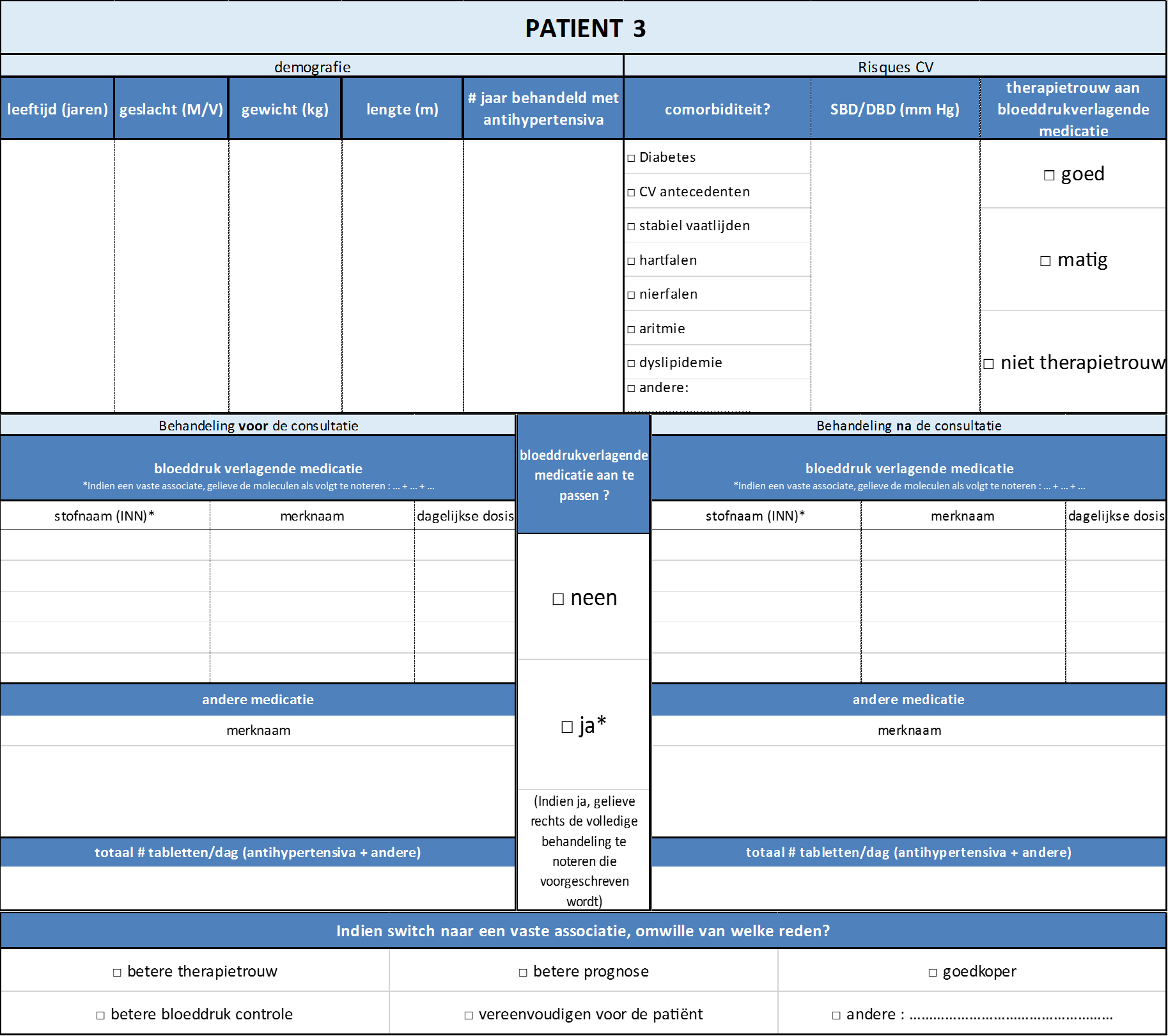


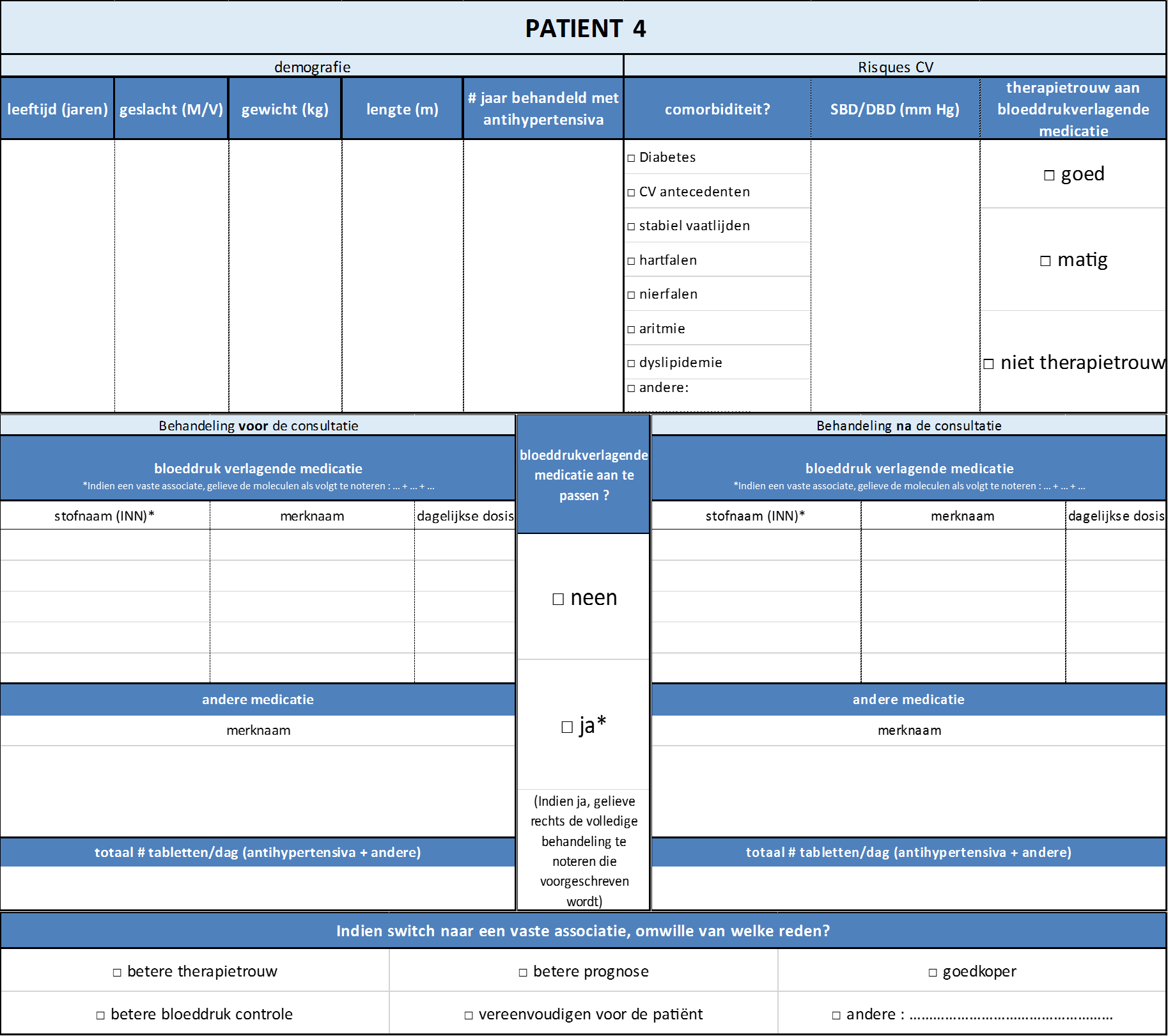


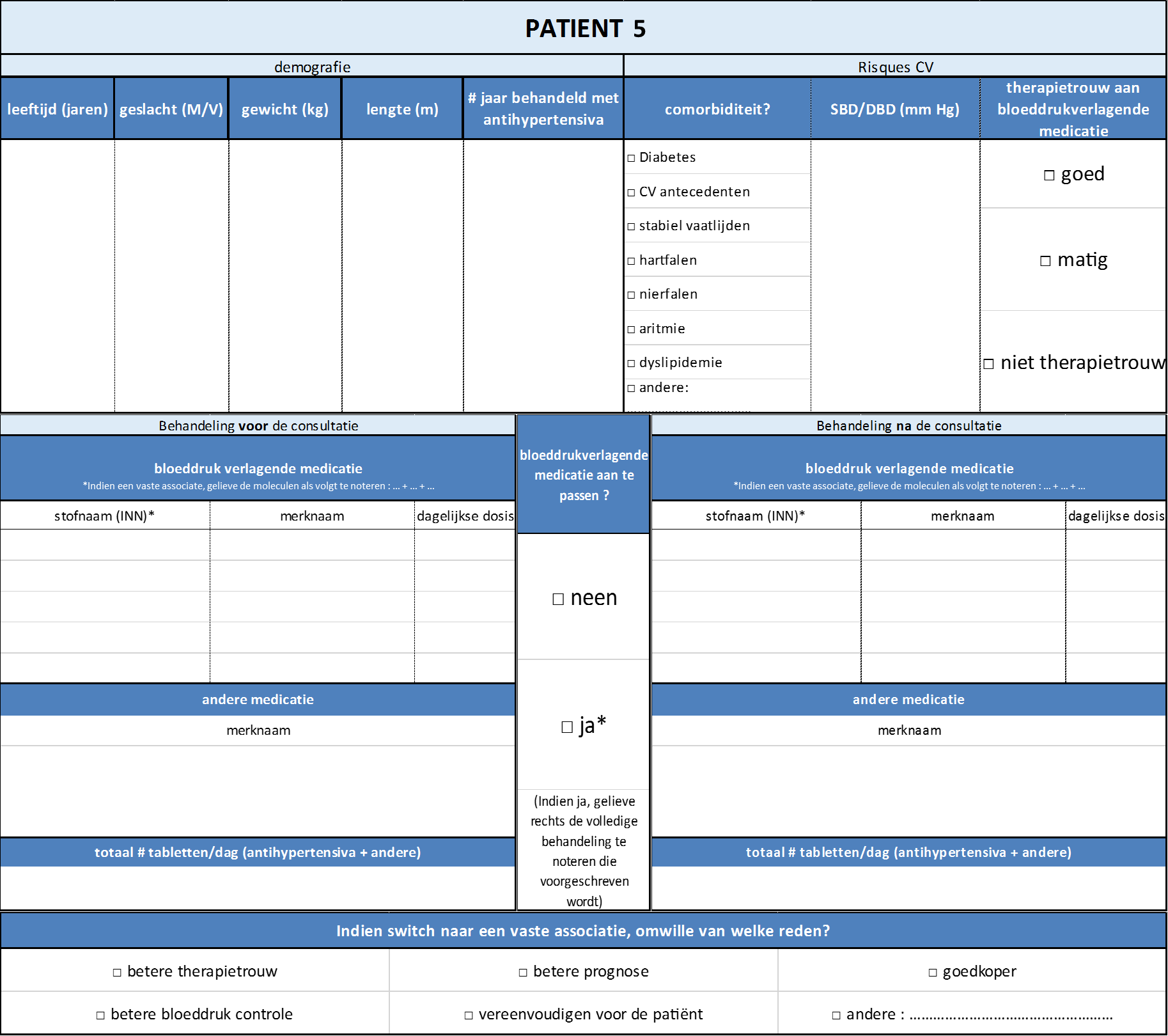


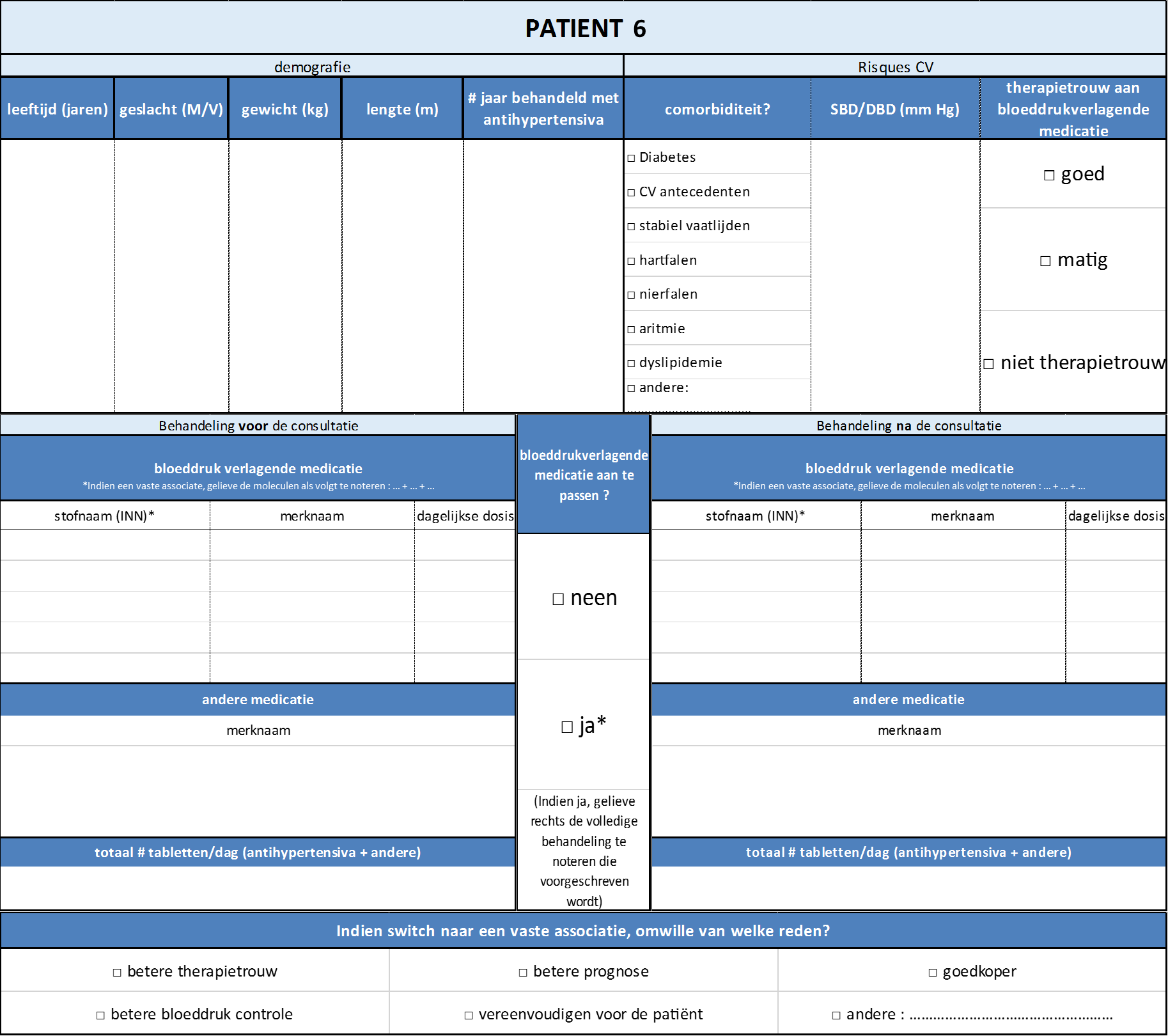


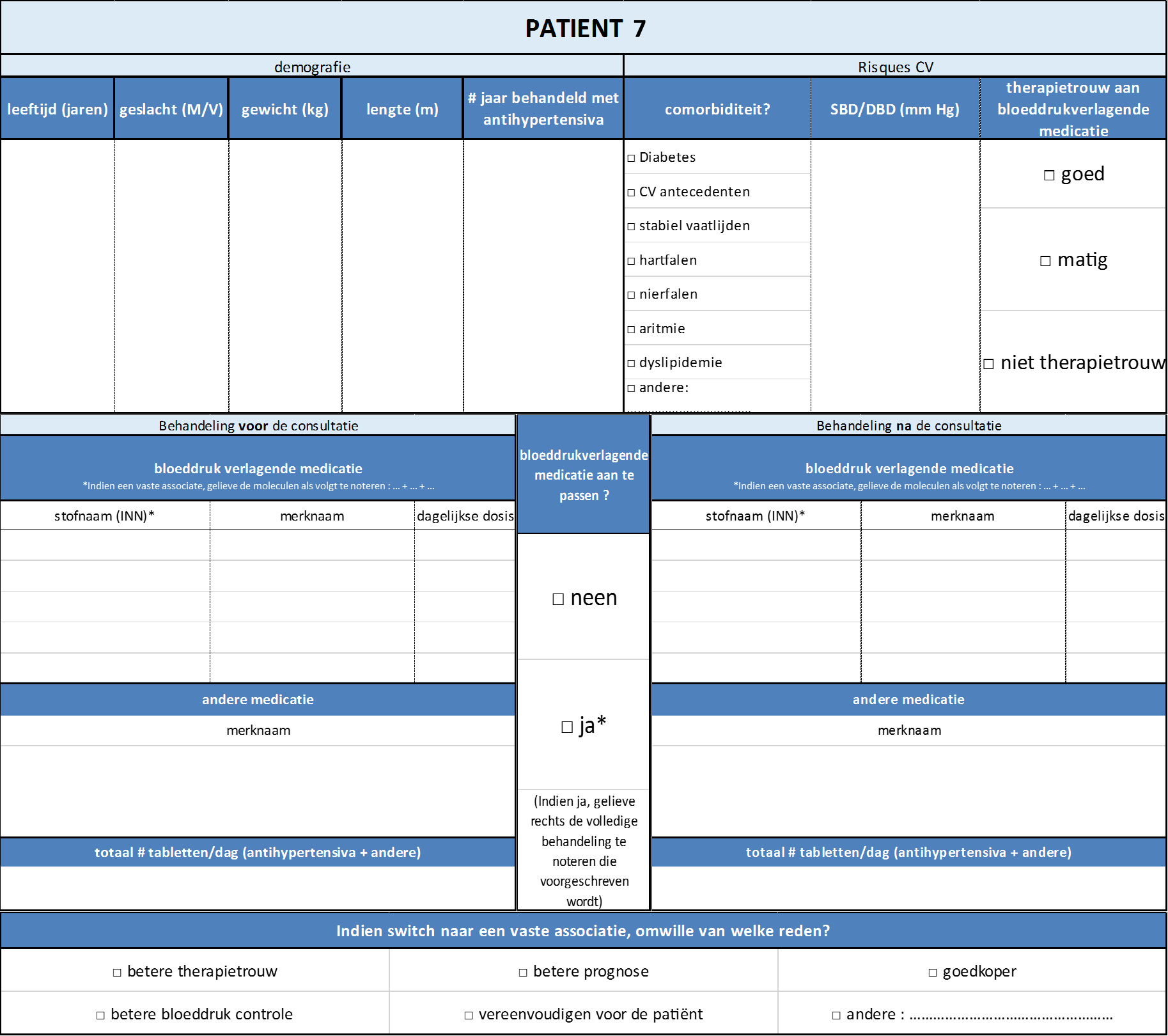


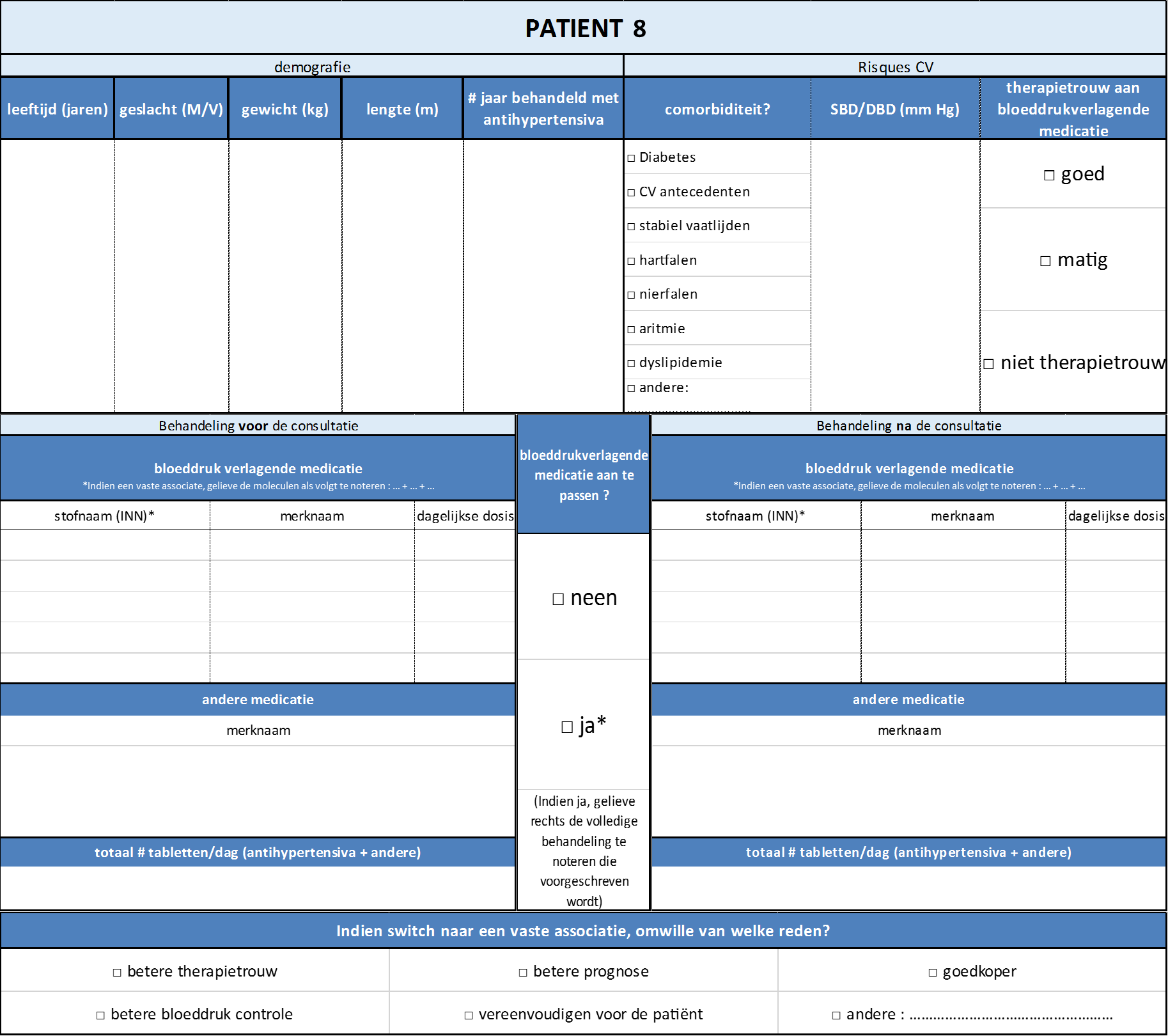


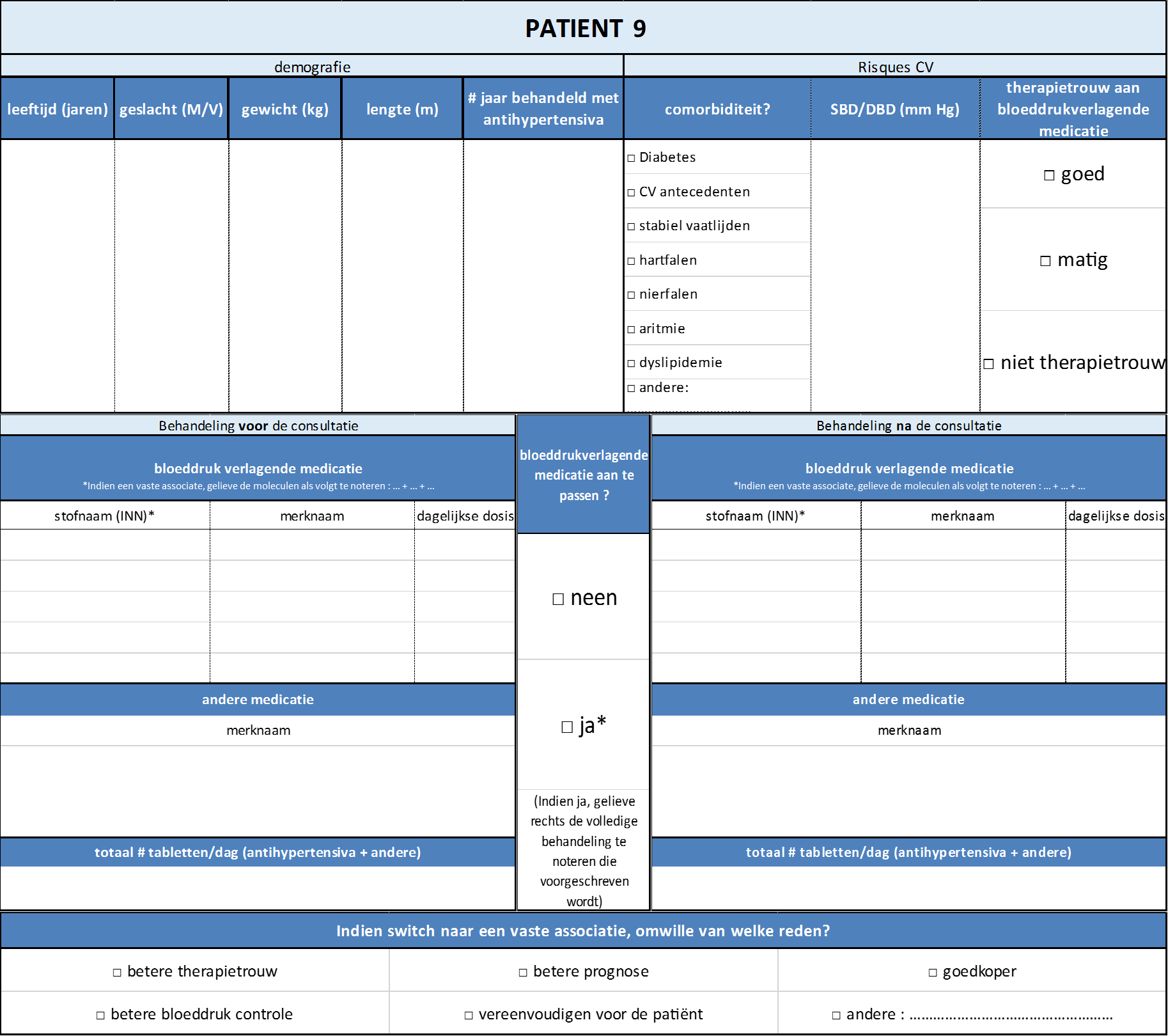


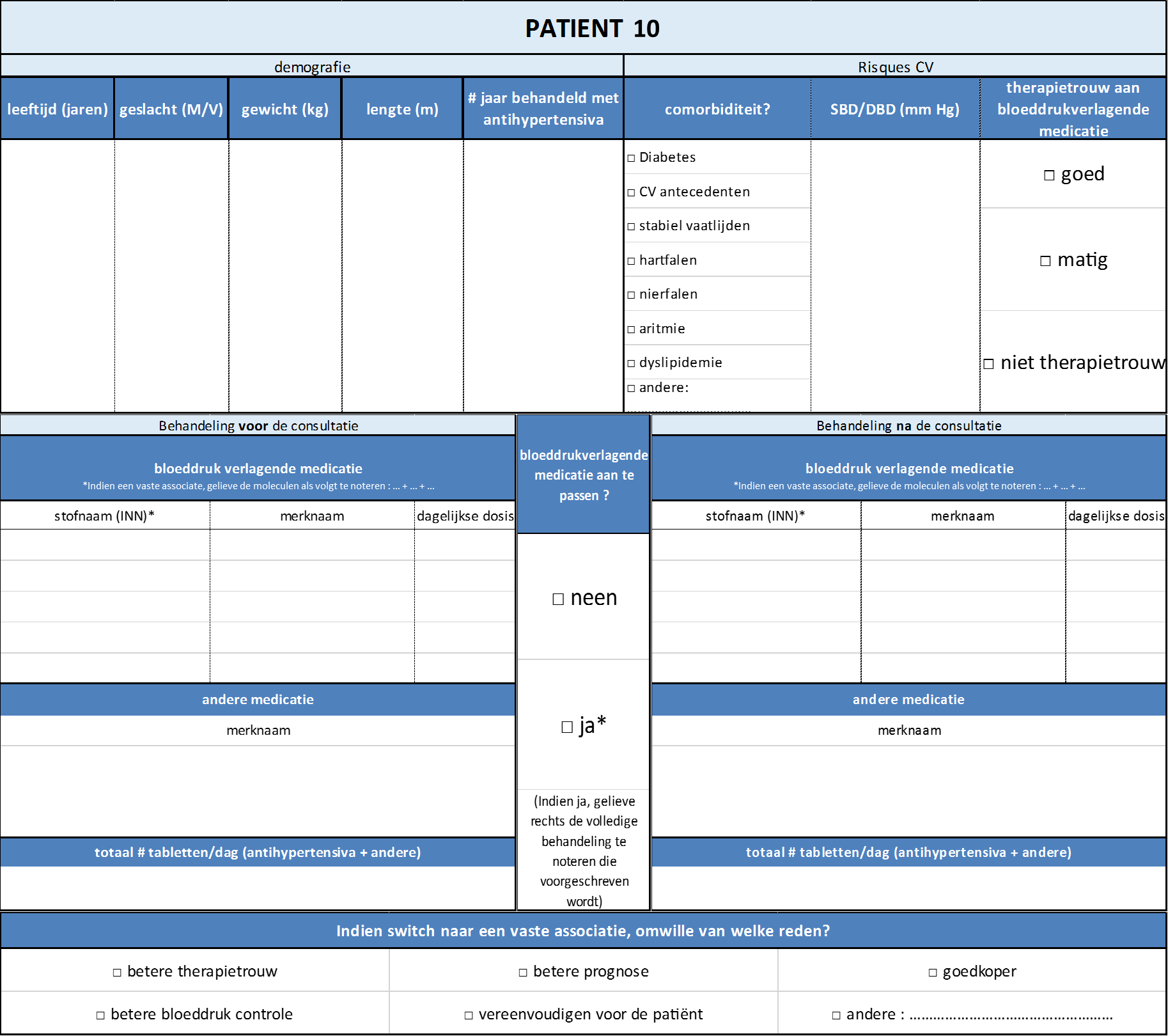


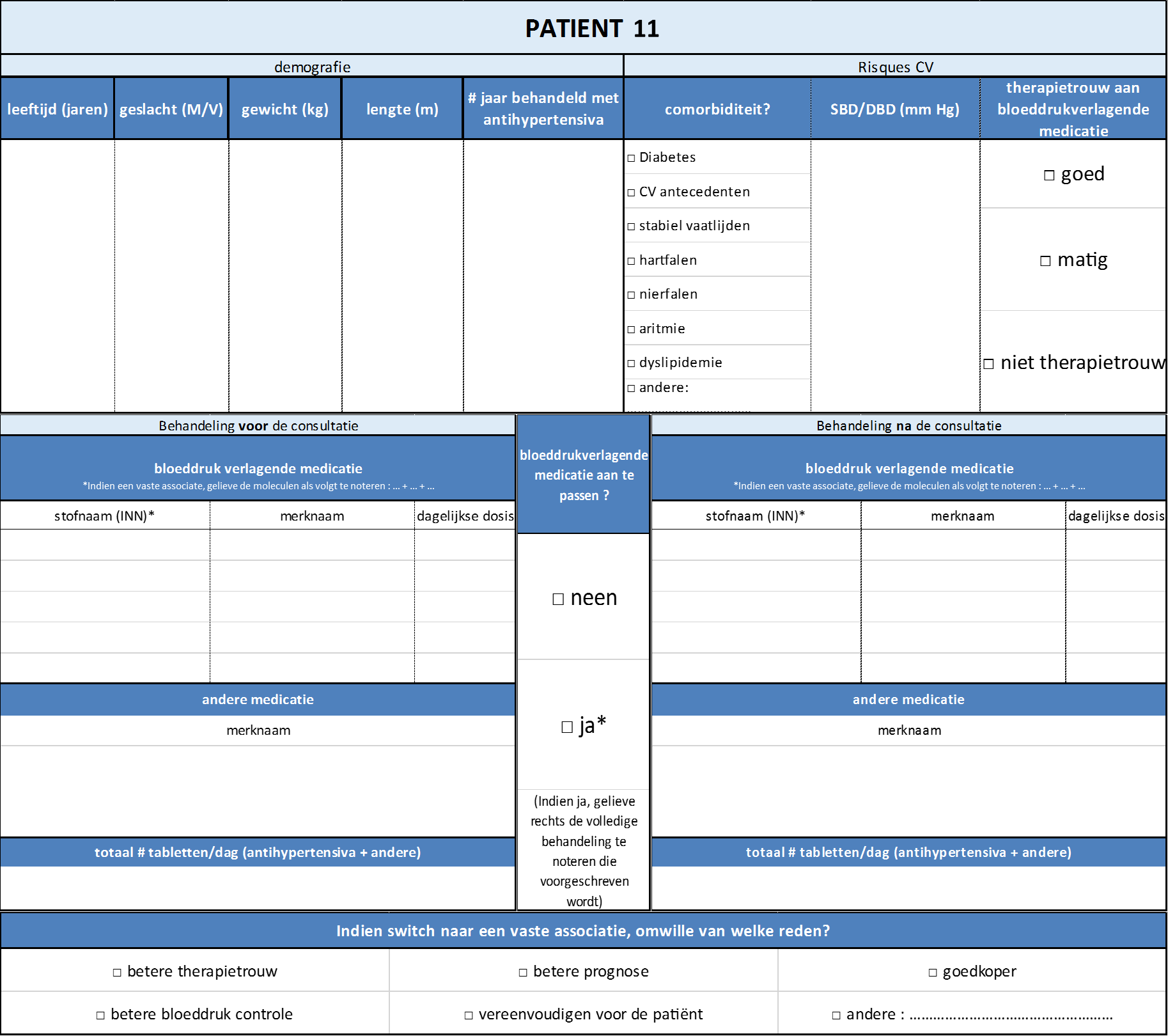


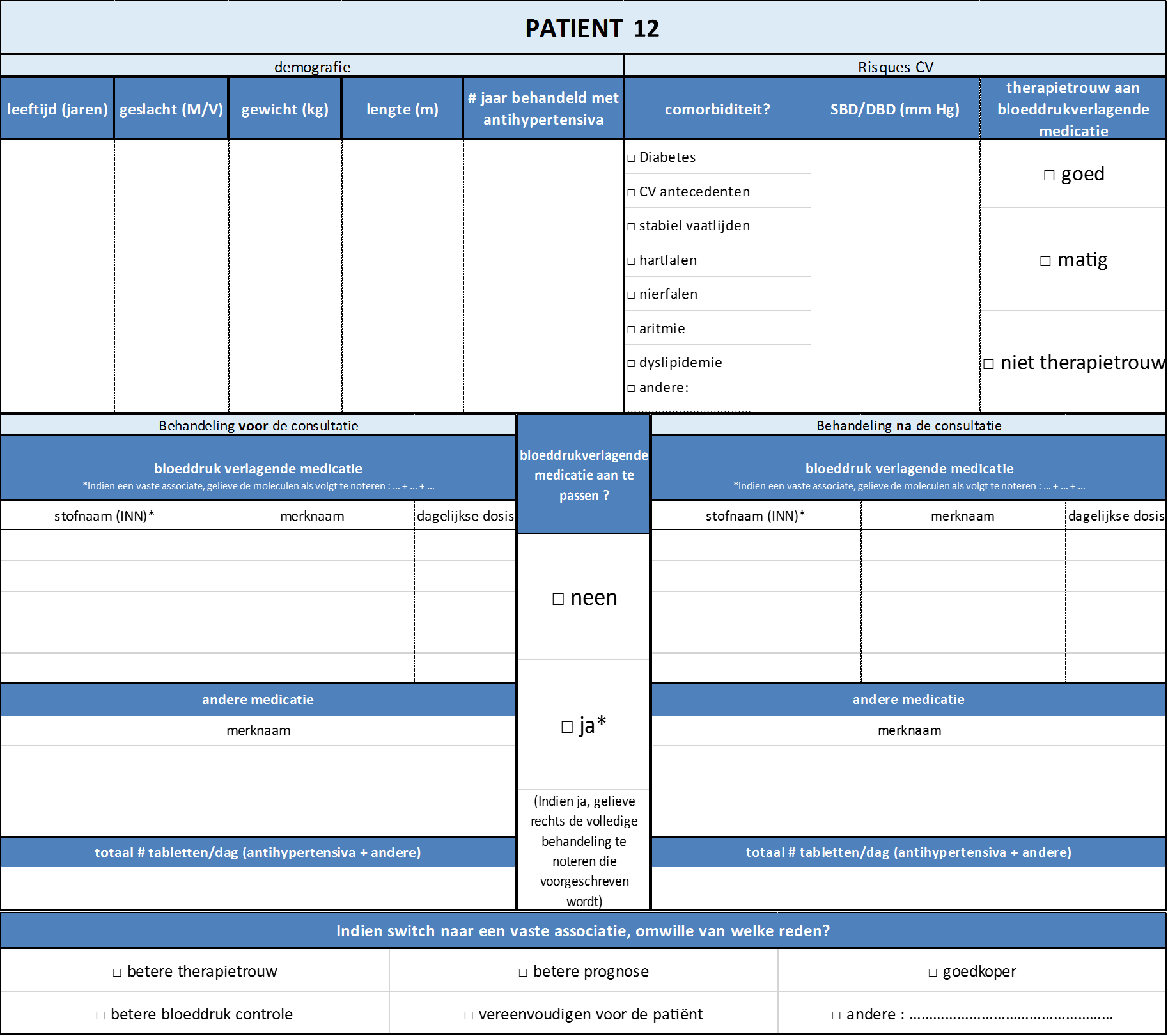


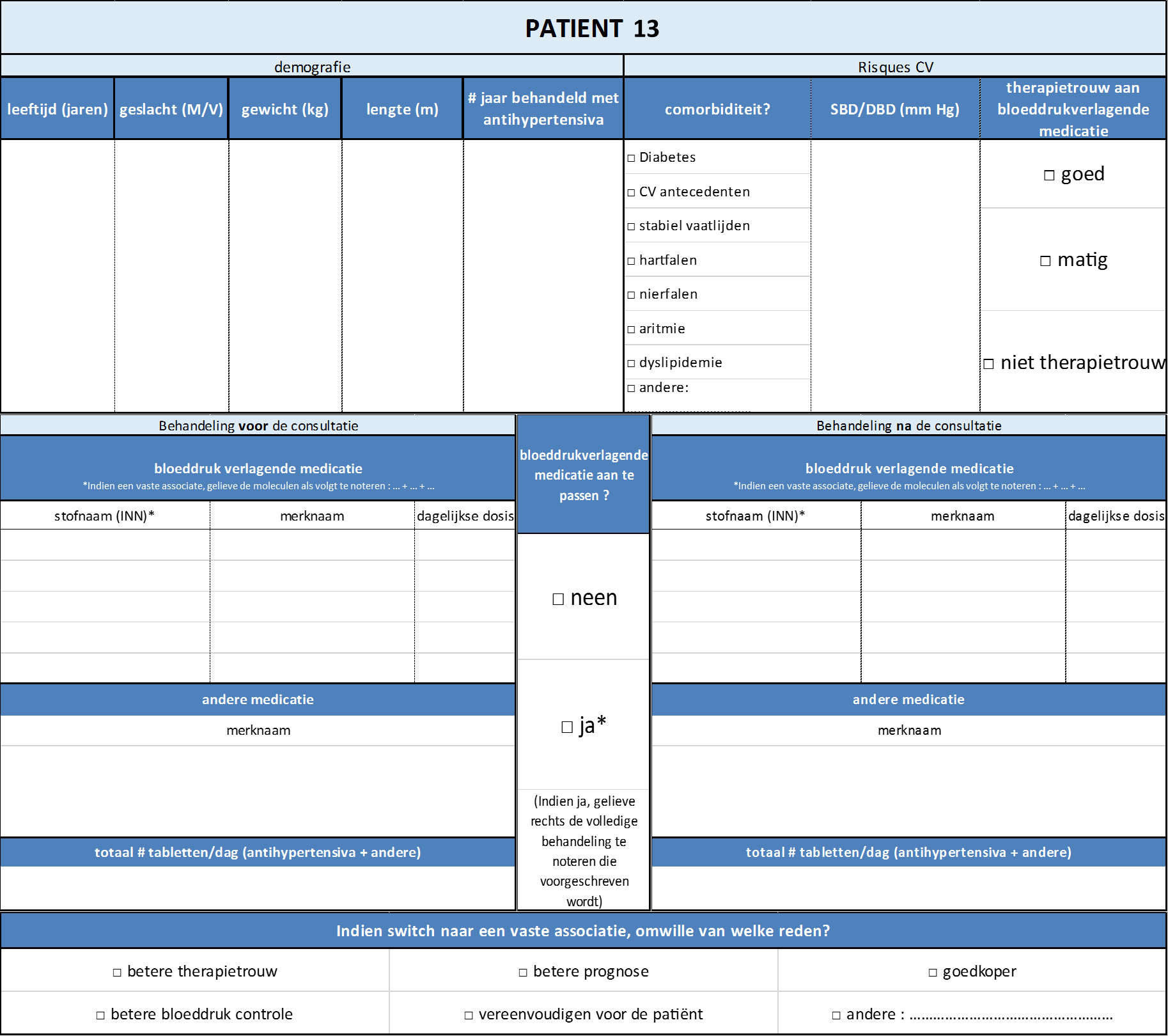


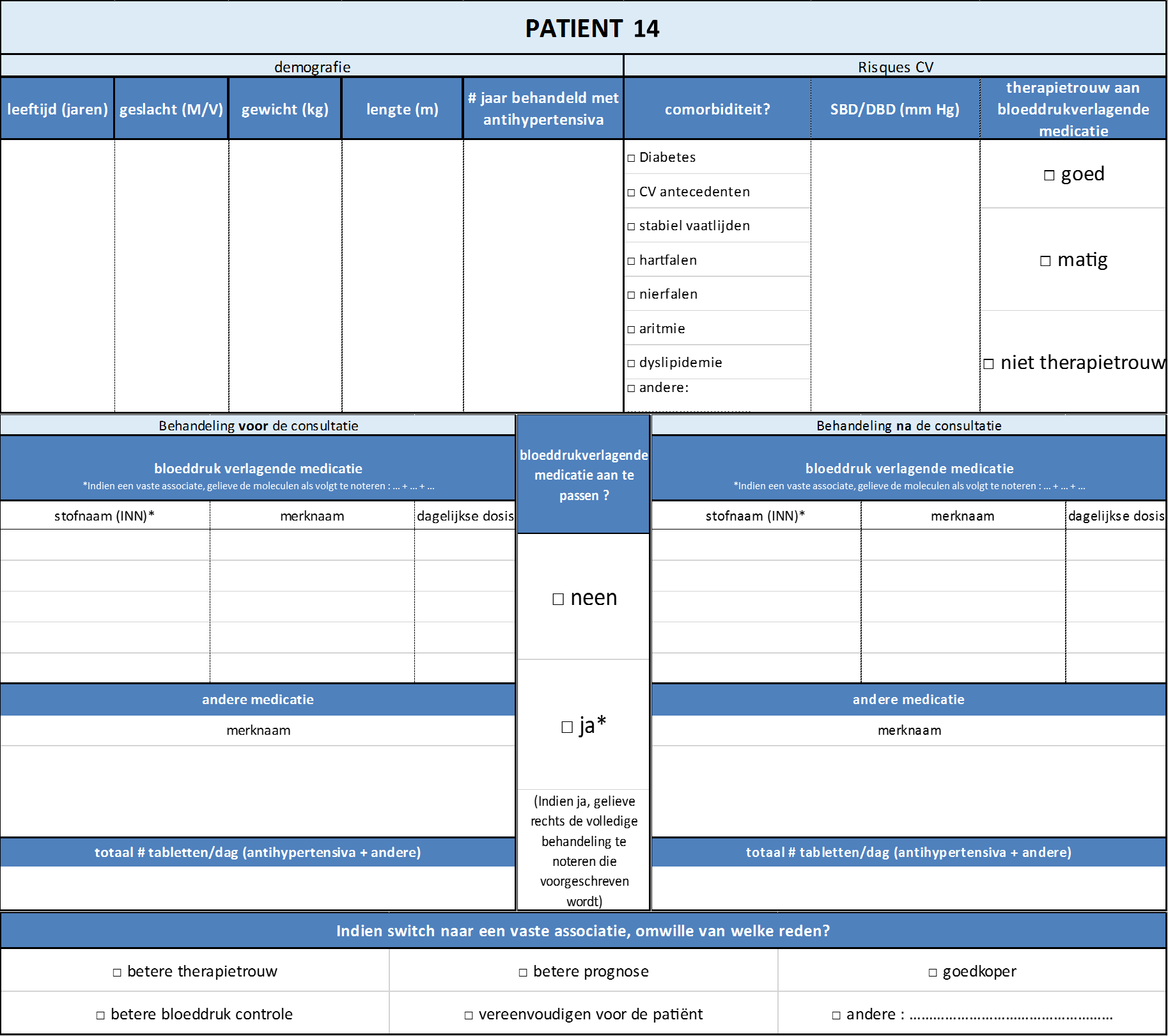


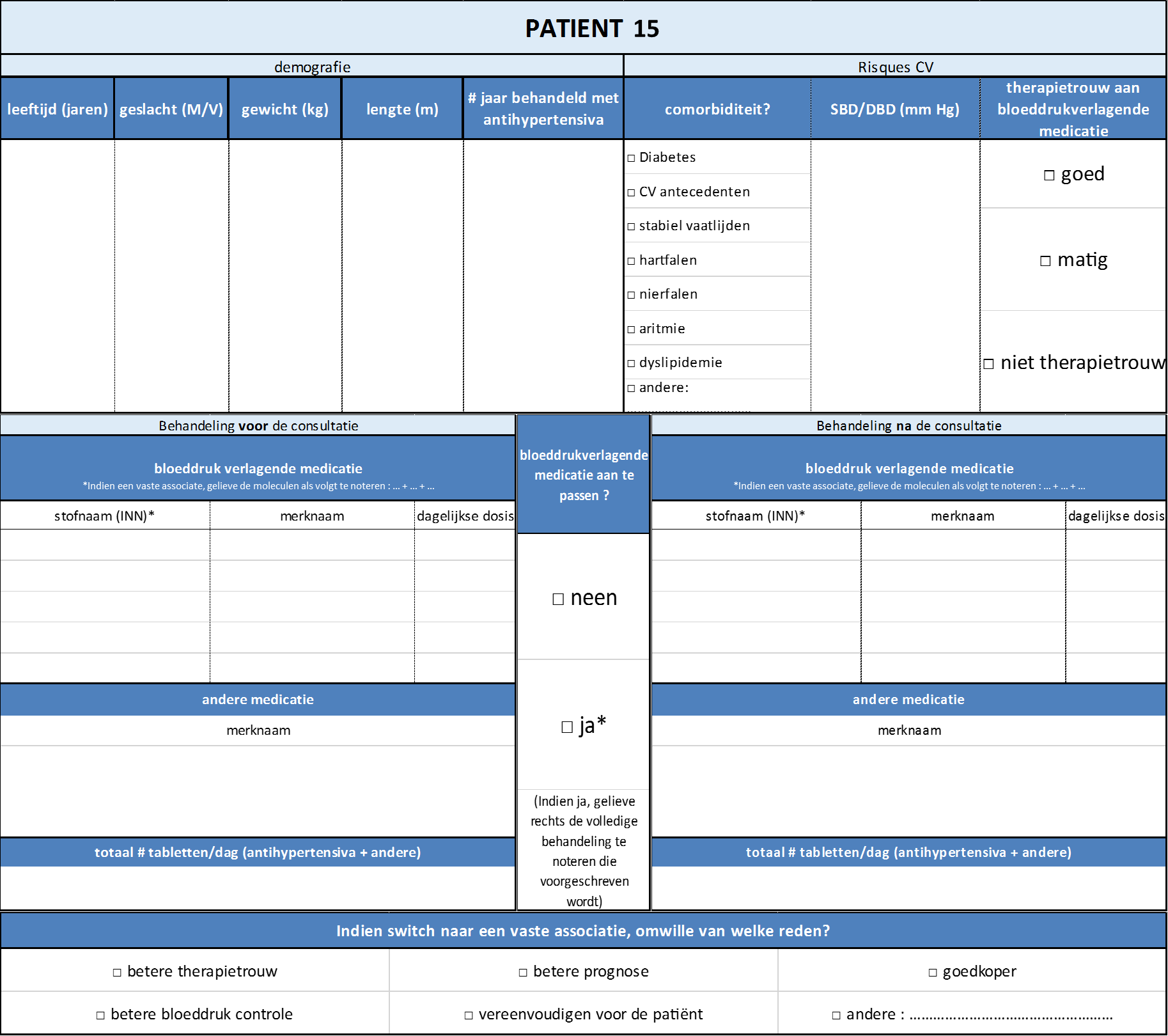

Supplement: S2 File — (DOC) [file pone.0248471.s003.doc]
