# Supplementary material for: Antihypertensive treatment in a general uncontrolled hypertensive population in Belgium and Luxembourg in primary care: Therapeutic inertia and treatment simplification. The SIMPLIFY study
Source: PLoS One. 2021 Apr 5;16(4):e0248471. doi: 10.1371/journal.pone.0248471 (PMC8021160; doi:10.1371/journal.pone.0248471)

**Study protocol**

**IC4-05150-023-BEL**

**Pharma.be VI 17/01/20/01**

***Scientific rational***

High blood pressure remains a major cause of death and one of the main modifiable risk factors for cardio-, cerebro- and renovascular diseases (1).

Despite the existence of several classes of effective antihypertensive drugs (diuretics, beta blockers, calcium channel blockers, ACE inhibitors and angiotensin receptor blockers), the treatment of many hypertensive patients remains suboptimal. It is estimated that at least half of hypertensive patients are unknown; that at least half of the patients treated remain uncontrolled; and that in the long term, half of patients on antihypertensive drugs do not adhere to treatment (2-4). This suboptimal treatment for hypertension has a significant impact on healthcare costs. It is to a large extent due to a certain therapeutic inertia on the part of the medical profession with regard to the adequate control of high blood pressure (5). Several factors seem to negatively influence the motivation of doctors to optimize the treatment of their hypertensive patients.

Hypertensive patients are often highly polymedicated, and are therefore treated with several tablets per day. Compliance has been shown to be highly dependent on the number of tablets taken per day. Patients treated with 3 different tablets per day are 74% less compliant than patients treated with only one tablet per day (6). Current recommendations from the European Hypertension Society and the European Cardiovascular Society advise the use of fixed combinations of antihypertensive drugs to improve compliance (7). In the case of bi- or triple therapies, European recommendations give preference to once-a-day treatments, since limiting the number of doses taken per day and the number of tablets promotes patient compliance and control of blood pressure. The recommendations of the European Society of Hypertension and the European Cardiology Society (7) also recommend adding a molecule to the treatment of uncontrolled hypertensive patients rather than increasing the dose. This is because the two treatments will act on different mechanisms, and therefore it is expected that this strategy will be more effective than increasing the dose.

In Belgium, few epidemiological data are available on the extent of this therapeutic inertia and on medical decision-making in the treatment of hypertension. This study aims to examine the extent to which Belgian general practitioners are motivated to intensify or simplify the treatment of uncontrolled hypertensive patients. In addition, it also examines what factors this medical decision sends to identify on which factors the various actors of the health system can act to improve the suboptimal treatment of hypertensive patients.

***2. Objective***

The objective of this study is to evaluate, in the current practice of general practitioners in Belgium, the treatment of uncontrolled hypertensive patients, as well as the motivation and the key factors encouraging doctors to simplify or intensify antihypertensive treatment, in terms of :

- blood pressure (systolic and diastolic) and blood pressure control

- compliance with antihypertensive treatment estimated by the doctor

- number / type of antihypertensive drugs used

- the number / type of drugs used

- the number and / or type of co-morbidities

- demographic factors (age, gender, BMI, etc.)

Expected added value

The completion of this study should allow:

- to know the degree of therapeutic inertia in the treatment of hypertension among general practitioners in Belgium;

- identify the factors motivating Belgian doctors to intensify or simplify antihypertensive treatment;

- to assess compliance with antihypertensive treatment in the population of uncontrolled hypertensive patients in Belgium.

***3. Design***

The methodology used will be a cross-sectional survey.

This study plan has the following characteristics:

- Only existing data usually collected in daily practice is collected. These routine data are not collected specifically to meet the purpose of the study.

- Data will be collected at a single precise moment in time, thus providing a cross-sectional view of the population studied. In this specific case, the study looks at the current situation (in 2017).

- The study is purely descriptive (statistical analysis can describe the current situation), but does not allow to study the evolution of this situation over time (only possible by a longitudinal study).

The methodology is perfectly suited to answer the questions asked.

Based on data already available and not involving the patients concerned, this type of study is considered to be "retrospective" within the meaning of paragraph 1.4 of the Guide to the evaluation of non-interventional studies of May 2008 (8), and does not fall within the scope of the law relating to experiments on human beings (May 7, 2004). In this context, the study obtained a prior visa from the visa office of pharma.be, following the recommendations made in its Code of ethics (March 23, 2012).

***4. Patients and investigators***

***4.1 Patients***

The patients targeted by the study are uncontrolled hypertensive patients (≥ 18 years old) (PA ≥140 / 90 mm Hg) already treated with at least 1 antihypertensive agent, and consulting a general practitioner in Belgium. Patients with secondary hypertension are excluded from the study.

The number of patients whose data will be collected is set at 4,725.

This number is not based on a statistical calculation, which is inherent to the type of study plan used, but is comparable to the current sample size for this type of epidemiological survey.

***4.2 Investigators***

The investigators targeted by the study are general practitioners, practicing in Belgium, who commonly see the type of patients targeted in their consultations.

The number of investigators required to collect data from 4,725 patients is estimated at 315, each collecting data from 15 eligible patients seen in consultation. These investigators will be recruited throughout Belgium, with a balanced geographic representation, over a period of 3 months (from March 16, 2017 to June 8, 2017). This is to allow Servier Benelux medical informants to set up meetings with the investigators for the initial visits; obtain their participation agreement and signature of the contract; and finally, provide them with the materials they need to initiate the study.

In order not to introduce patient selection bias, investigators are asked to include the last 15 consecutive patients meeting the criteria of the study who recently attended their consultation.

***5. Data collected***

For each eligible patient, the following existing data will be collected by the investigator on an observation form. Data is collected from the patient's medical records. There is no plan to make future observation (consultation) contacts with patients.

- Patient number in the study (from 1 to 15);

- Age, sex, height and weight;

- Presence or not of comorbidities (diabetes, cardiovascular history, stable coronary disease, heart failure, renal failure, arrhythmia, dyslipidemia, other);

- Systolic / diastolic blood pressure (mmHg);

- Patient compliance with antihypertensive treatment estimated by the doctor (good, average, patient not compliant);

- Treatment before consultation: antihypertensive agent (s) used (brand name, INN, dose), other drug treatments, total number of tablets per day;

- Medical decision during the consultation (adapt the treatment: yes or no);

- Treatment after consultation: antihypertensive agent (s) used (brand name, INN, dose), type of combination (free or fixed), other drug treatments, total number of tablets per day;

- Motivation for the use of fixed associations (if applicable);

In addition to patient data, the observation form will also collect the name of the investigator, his address, his signature and his stamp. This in order to be able to contact him if necessary, and as a guarantee of authenticity.

The observation form mentions the visa number of pharma.be as well as the name of the contact person at Servier Benelux, in order to allow the request for additional information if necessary.

The data necessary for the study will be collected during a period of 4 months following the agreement of the investigator to participate in the study, in order to give these busy generalists the time necessary to identify the 15 eligible patients and fill out the corresponding observation forms. Thereafter, Servier Benelux medical representatives will have 2 months to collect the observation forms. As the deadline for recruiting investigators is June 8, 2017, all data collected should be available on November 8, 2017, the end of study date. The data collected through this cross-sectional study is the property of Servier Benelux. The publication of data from this database can only be done with the agreement of Servier Benelux.

If the investigator, as part of the research of the data to be collected for the study in a patient's file, identifies an undesirable effect potentially linked to the taking of one of Servier Benelux's medicines and which has not (yet) been notified to Servier Benelux, he is asked to report it as soon as possible by completing the adverse reaction report form and to send it immediately to the pharmacovigilance manager of Servier Benelux, Dr Xavier Pottier (fax: 025294389, e-mail: [pharmacovigilance@be.netgrs.com](../pharmacovigilance@be.netgrs.com), tel: 025294311). If necessary, the pharmacovigilance manager of Servier Benelux will contact the investigator to obtain more details.

This adverse reaction report form is also to be used for the following cases:

- All adverse events

- An exposure during pregnancy or breastfeeding,

- Overdose (intentional or accidental), abuse or misuse

- Off-label use (off-indication)

- A professional exhibition,

- Lack of efficiency

***6. Analysis of results***

After a quality control, the data from all correctly completed observation forms will be entered into an Excel table allowing the following descriptive statistical analyzes:

- Population studied: number of patients, distribution according to demographic data (age, sex, BMI), cardiovascular risk (co-morbidities, systolic and diastolic blood pressure, compliance with antihypertensive treatment estimated by the doctor) and treatment (total number of antihypertensive agents used, type of antihypertensive drugs used, and total number of drugs used)

- Control of systolic and diastolic blood pressure: total population vs population at risk, association with compliance with antihypertensive treatment estimated by the doctor, relationship with the total number of antihypertensive agents used, the types of antihypertensive agents and the total number of drugs used .

- Medical decision: relationship between intensification or simplification of antihypertensive treatment and demographic factors, risk factors, systolic and diastolic blood pressure, compliance with antihypertensive treatment estimated by the doctor, the total number of antihypertensive agents used, types of antihypertensives and the total number of drugs used.

- Motivation to use fixed combinations in the treatment of hypertension.

***7. Study report and publication***

After analyzing the data and interpreting the results, a study report will be drawn up by the scientific department of Servier Benelux. Publication in a scientific journal will also be considered.

This report (or publication) will be given to each of the investigators who participated in the study. He (she) will also be made available to the bodies of pharma.be set out in article 52, §1 of its Code of ethics of March 23, 2012.

***8. Quality management***

The study protocol was developed in collaboration with the scientific department of Servier Benelux, which then approved it, and which will ensure the smooth running of the study.

The study received a prior visa from the visa office of pharma.be, following the recommendations made in its Code of ethics (March 28, 2014).

In order to guarantee the quality of the data collected, a quality control will be organized, which will be adapted to the quality risks inherent in the observational methodology of the study. To this end, the observation forms will be verified by the scientific service of Servier Benelux in a sample of 5% of the investigators who participated in the study.

Data management, statistical analyzes and the drafting of the study report will be carried out by the scientific department of Servier Benelux.

The role of Servier Benelux medical informants will be limited to the following two actions:

- Initiation visit: presentation of the study, submission of the protocol and observation form, signature of the contract with the investigator.

- Closing visit: resumption of the completed observation forms.

***9. References***

1. Lewington S et al. Age-specific relevance of ususal blood pressure to vascular mortality: a meta-analysis of individual data for one million adults in 61 prospective studies. Lancet 2002; 360: 903-913
2. Duprez et al. Prevalence of hypertension in the adult population of Belgium: report of a worksite study, Attention Hypertension. J Hum Hypertens 2002; 16: 47-52
3. Fagard et al. Treatment and blood pressure control in isolated systolic hypertension vs diastolic hypertension in primary care. Journal of Hypertension 2002; 20: 1297-1302
4. Erdine S. How well is hypertension controlled in Europe? ESH Newsletter 2011; 12:5-6
5. Redon et al. Why in 2016 are patients with hypertension not 100% controlled? A call to action. Journal of Hypertension 2016;34:1480-1488
6. Xie et al., A medication adherence and persistence comparison of hypertensive patients treated with single-, double- and triple-pill combination therapy Curr Med Res & Op. 2014;30:15-22.
7. Mancia G, Fagard R et al. 2013 ESH/ESC Guidelines for the management of arterial hypertension. Journal of Hypertension 2013, 31: 1281-1357.
8. Bogaert M et al. Guide d’évaluation des études non interventionnelles. http://www.fagg-afmps.be/fr/humain/medicaments/medicaments/recherche_developpement/comite_d_ethique/

***10.* *Signatures***

**Investigator  Servier Benelux**

Name : Name : Van Nieuwenhuyse Bregt

Signature : Signature :


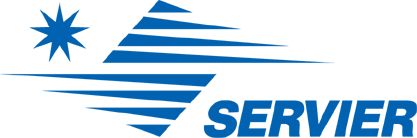

Supplement: S4 File — (DOC) [file pone.0248471.s005.doc]
