# Supplementary material for: Antihypertensive treatment in a general uncontrolled hypertensive population in Belgium and Luxembourg in primary care: Therapeutic inertia and treatment simplification. The SIMPLIFY study
Source: PLoS One. 2021 Apr 5;16(4):e0248471. doi: 10.1371/journal.pone.0248471 (PMC8021160; doi:10.1371/journal.pone.0248471)

**Étude observationnelle transversale évaluant la motivation et les facteurs clés incitant les médecins généralistes à SIMPLIFIER ou à intensifier le traitement antihypertenseur dans la population générale d’hypertendus belges qui sont non contrôlés et traités par au moins 1 agent antihypertenseur**

**Protocole d’étude**

**IC4-05150-023-BEL**

**Pharma.be VI 17/01/20/01**

***Rationnel scientifique***

L’hypertension artérielle reste une cause majeure de mortalité et un des principaux facteurs de risque modifiables des maladies cardio-, cérébro- et rénovasculaires (1).

Malgré l'existence de plusieurs classes de médicaments antihypertenseurs efficaces (diurétiques, bêta-bloquants, inhibiteurs calciques, inhibiteurs de l'ECA et antagonistes des récepteurs de l'angiotensine), le traitement de nombreux patients hypertendus reste sous-optimal. On estime qu’au moins la moitié des patients hypertendus est méconnue ; qu’au moins la moitié des patients traités restent non-contrôlés ; et qu’à long terme, la moitié des patients sous antihypertenseurs n’adhère pas au traitement (2-4). Ce traitement sous-optimal de l'hypertension a un impact significatif sur les coûts des soins de santé. Il est dans une large mesure imputable à une certaine inertie thérapeutique du corps médical quant au contrôle adéquat d’une pression artérielle élevée (5). Plusieurs facteurs semblent influencer négativement la motivation des médecins à optimiser le traitement de leurs patients hypertendus.

Les patients hypertendus sont souvent fortement polymédiqués, et sont donc traités avec plusieurs comprimés par jour. Il est démontré que la compliance est fortement dépendante du nombre de comprimés pris par jour. Des patients traités avec 3 comprimés différents par jour sont 74% moins compliants que des patients traités avec un seul comprimé par jour (6). Les recommandations actuelles de la Société européenne d'hypertension et de la Société européenne de cardiologie conseillent d'utiliser des associations fixes de médicaments antihypertenseurs afin d’améliorer la compliance (7). Dans le cas des bi- ou trithérapies, les recommandations européennes donnent la préférence aux traitements en une prise par jour, car la limitation du nombre de prises par jour et du nombre de comprimés favorise la compliance du patient et le contrôle de la pression artérielle. Les recommandations de l'European Society of Hypertension et la Société européenne de cardiologie (7) préconisent également d'ajouter une molécule au traitement des patients hypertendus non contrôlés plutôt que d'augmenter la dose. Cela parce que les deux traitements vont agir sur des mécanismes différents, et qu’on s’attend à donc à ce que cette stratégie soit plus efficace que l'augmentation de la dose.

En Belgique, peu de données épidémiologiques sont disponibles quant à l’ampleur de cette inertie thérapeutique et quant à la prise de décision médicale dans le traitement de l'hypertension. Cette étude vise à examiner dans quelle mesure les médecins généralistes belges sont motivés à intensifier ou simplifier le traitement des patients hypertendus non contrôlés. En outre, elle examine également quels facteurs cette décision médicale envoie pour identifier sur quels facteurs les différents acteurs du système de santé peuvent agir pour améliorer le traitement sous-optimal des patients hypertendus.

***2. Objectif***

L’objectif de la présente étude est d’évaluer, dans la pratique courante des médecins généralistes en Belgique, le traitement des patients hypertendus non contrôlés, ainsi que la motivation et les facteurs clés incitant les médecins à simplifier ou à intensifier le traitement antihypertenseur, en fonction de :

- la pression artérielle (systolique et diastolique) et le contrôle de la pression artérielle
- la compliance au traitement antihypertensif estimé par le médecin
- le nombre / le type des médicaments antihypertenseurs utilisés
- le nombre / le type des médicaments utilisés
- le nombre et/ou le type des comorbidités
- les facteurs démographiques (âge, sexe, BMI,…)

*Valeur ajoutée attendue*

La réalisation de cette étude devrait permettre :

- de connaître le degré de l’inertie thérapeutique dans le traitement de l’hypertension chez les médecins généralistes en Belgique ;
- d’identifier les facteurs motivant les médecins belges à intensifier ou simplifier le traitement antihypertenseur ;
- d’estimer la compliance au traitement antihypertensif dans la population des patients hypertendus non contrôlés en Belgique.

***3. Design***

La méthodologie utilisée sera celle d’une étude observationnelle transversale *(cross-sectional survey)*.

Ce plan d’étude a les caractéristiques suivantes :

- Seules des données existantes recueillies habituellement en pratique quotidienne sont collectées. Ces données de routine ne sont pas recueillies spécifiquement pour répondre à l’objectif de l’étude.
- Les données seront collectées à un seul instant précis dans le temps, permettant ainsi d’avoir une vue transversale sur la population étudiée. Dans ce cas précis, l’étude s’intéresse à la situation actuelle (en 2017).
- L’étude est purement descriptive (l’analyse statistique peut décrire la situation actuelle), mais ne permet pas d’étudier l’évolution de cette situation dans le temps (seulement possible par une étude longitudinale).

La méthodologie est parfaitement adaptée pour répondre aux questions posées.

Partant de données déjà disponibles et n’impliquant pas les patients concernés, ce type d’étude est considéré comme « rétrospective » dans le sens du paragraphe 1.4 du Guide d’évaluation des études non-interventionnelles de mai 2008 (8), et ne relève donc pas du champ d’application de la loi relative aux expérimentations sur la personne humaine (7 mai 2004). Dans ce cadre, l’étude a obtenu un visa préalable du Bureau des visas de pharma.be, suivant les recommandations émises dans son Code de déontologie (23 mars 2012).

***4. Patients et investigateurs***

*4.1 Patients*

Les patients visés par l’étude sont des patients hypertendus (≥ 18 ans) non contrôlés (PA ≥140/90 mm Hg) déjà traités avec au moins 1 antihypertenseur, et consultant un médecin généraliste en Belgique. Les patients avec une hypertension secondaire sont exclus de l’étude.

Le nombre de patients dont les données seront collectées est fixé à 4725.

Ce nombre n’est pas basé sur un calcul statistique, ce qui est inhérent au type de plan d’étude utilisé, mais est comparable à la taille d’échantillon courante pour ce type d’enquête épidémiologique.

*4.2 Investigateurs*

Les investigateurs visés par l’étude sont des médecins généralistes, pratiquant en Belgique, et qui voient couramment le type de patients visés dans leurs consultations.

Le nombre d’investigateurs nécessaire pour collecter les données de 4725 patients est estimé à 315, chacun collectant les données de 15 patients éligibles vus en consultation. Ces investigateurs seront recrutés dans l’ensemble de la Belgique, avec une représentation géographique équilibrée, au cours d’une période de 3 mois (du 16 mars 2017 au 8 juin 2017). Cela afin de permettre aux informateurs médicaux de Servier Benelux de mettre sur pied les rendez-vous avec les investigateurs pour les visites initiales ; d’obtenir leur accord de participation et leur signature du contrat ; et enfin, de leur remettre le matériel nécessaire pour l’initiation de l’étude.

Afin de ne pas introduire de biais de sélection de patients, il est demandé aux investigateurs d’inclure les 15 *derniers* patients *consécutifs* répondant aux critères de l’étude qui se sont présentés récemment à leur consultation.

***5. Données collectées***

Pour chaque patient éligible, les données existantes suivantes seront collectées par l’investigateur sur un formulaire d’observation. Les données sont recueillies sur base des dossiers médicaux des patients. Il n'y a pas de plan de faire des futurs contacts d’observations (consultation) avec les patients.

- N° du patient dans l’étude (de 1 à 15) ;
- Âge, sexe, taille et poids ;
- Présence ou non de comorbidités (diabète, antécédent cardiovasculaire, maladie coronaire stable, insuffisance cardiaque, insuffisance rénale, arythmie, dyslipidémie, autre) ;
- Pression artérielle systolique/diastolique (mmHg) ;
- Compliance du patient au traitement antihypertensif estimée par le médecin (bonne, moyenne, patient non compliant) ;
- Traitement avant la consultation : antihypertenseur(s) utilisé(s) (nom de marque, INN, dose), autres traitements médicamenteux, nombre total de comprimés par jour ;
- Décision médicale au cours de la consultation (adapter le traitement : oui ou non) ;
- Traitement après la consultation: antihypertenseur(s) utilisé(s) (nom de marque, INN, dose), type d’association (libre ou fixe), autres traitements médicamenteux, nombre total de comprimés par jour ;
- Motivation pour l’usage d’associations fixes (si applicable) ;

En plus des données des patients, le formulaire d’observation récoltera également le nom de l’investigateur, son adresse, sa signature et son cachet. Cela afin de pouvoir le contacter si nécessaire, et comme garantie d’authenticité.

Le formulaire d’observation mentionne le numéro de visa de pharma.be ainsi que le nom de la personne de contact chez Servier Benelux, afin de permettre la demande d’informations complémentaires en cas de besoin.

Les données nécessaires à l’étude seront collectées au cours d’une période de 4 mois suivant l’accord de l’investigateur de participer à l’étude, afin de laisser à ces généralistes à l’emploi du temps chargé le temps nécessaire pour identifier les 15 patients éligibles et pour remplir les formulaires d’observation correspondants. Ensuite, les visiteurs médicaux de Servier Benelux auront 2 mois pour recueillir les formulaires d’observation. Comme la date limite de recrutement des investigateurs est le 8 juin 2017, toutes les données collectées devraient être disponibles le 8 novembre 2017, date de fin d’étude. Les données récoltées grâce à cette étude transversale sont la propriété de Servier Benelux. La publication de données issue de cette banque de données ne pourra se faire qu’après accord de Servier Benelux.

Si l’investigateur, dans le cadre de la recherche des données à récolter pour l’étude dans le dossier d’un patient, identifie un effet indésirable potentiellement lié à la prise d’un des médicaments de Servier Benelux et qui n’a pas (encore) été notifié à Servier Benelux, il lui est demandé de le rapporter dans les plus brefs délais en complétant le formulaire de Rapport d’effet indésirable et de l’envoyer immédiatement au responsable de la pharmacovigilance de Servier Benelux, le Dr Xavier Pottier (fax : 025294389 , e-mail : [pharmacovigilance@be.netgrs.com](mailto:pharmacovigilance@be.netgrs.com), tél : 025294311). Si besoin, le responsable de la pharmacovigilance de Servier Benelux contactera l’investigateur afin d’obtenir plus de détails.

Ce formulaire de rapport d’effets indésirables est également à utiliser pour les cas suivant :

- Tous événements indésirables
- Une exposition pendant la grossesse ou l’allaitement,
- Un surdosage (intentionnel ou accidentel), abus ou mésusage
- Une utilisation off-label (hors-indication)
- Une exposition professionnelle,
- Un manque d’efficacité

***6. Analyse des résultats***

Après un contrôle de qualité, les données de tous les formulaires d’observation correctement remplis seront introduites dans un tableau Excel permettant les analyses statistiques descriptives suivantes :

- Population étudiée : nombre de patients, répartition suivant les données démographiques (age, sexe, BMI), risque cardiovasculaire (co-morbidités, pression artérielle systolique et diastolique, compliance au traitement antihypertensif estimée par le médecin) et traitement (nombre total des antihypertenseurs utilisés, type des antihypertenseurs utilisé, et nombre total des médicaments utilisés)
- Contrôle de la pression artérielle systolique et diastolique : population totale vs population à risque, association avec la compliance au traitement antihypertensif estimée par le médecin, relation avec le nombre total des antihypertenseurs utilisés, les types d’antihypertenseurs et le nombre total des médicaments utilisés.
- Décision médicale : relation entre l’intensification ou la simplification du traitement antihypertenseur et les facteurs démographiques, les facteurs de risque, la pression artérielle systolique et diastolique, la compliance au traitement antihypertensif estimée par le médecin, le nombre total des antihypertenseurs utilisés, les types d’antihypertenseurs et le nombre total des médicaments utilisés.
- Motivation pour utiliser des associations fixes dans le traitement d’hypertension.

***7. Rapport d’étude et publication***

Après l’analyse des données et l’interprétation des résultats, un rapport d’étude sera rédigé par le service scientifique de Servier Benelux. Une publication dans une revue scientifique sera également envisagée.

Ce rapport (ou publication) sera remis(e) à chacun des investigateurs ayant participé à l’étude. Il (elle) sera également mis(e) à la disposition des organes de pharma.be énoncés à l’article 52, §1 de son Code de déontologie dd 23 mars 2012.

***8. Gestion de qualité***

Le protocole d’étude a été élaboré en collaboration avec le service scientifique de Servier Benelux, qui l’a ensuite approuvé, et qui veillera au bon déroulement de l’étude.

L’étude a reçu un visa préalable du Bureau des visas de pharma.be, suivant les recommandations émises dans son Code de déontologie (28 mars 2014).

Afin de garantir la qualité des données récoltées, un contrôle de qualité sera organisé, qui sera adapté aux risques de qualité inhérents à la méthodologie observationnelle de l’étude. À cette fin, les formulaires d’observation seront vérifiés par le service scientifique de Servier Benelux chez un échantillon de 5% des investigateurs ayant participé à l’étude.

La gestion des données, les analyses statistiques, ainsi que la rédaction du rapport d’étude seront réalisées par le service scientifique de Servier Benelux.

Le rôle des informateurs médicaux de Servier Benelux sera limité aux deux actions suivantes :

- Visite d’initiation : présentation de l’étude, remise du protocole et du formulaire d’observation, signature du contrat avec l’investigateur.
- Visite de clôture : reprise des formulaires d’observations complétés.

***9. Références***

1. Lewington S et al. Age-specific relevance of ususal blood pressure to vascular mortality: a meta-analysis of individual data for one million adults in 61 prospective studies. Lancet 2002; 360: 903-913
2. Duprez et al. Prevalence of hypertension in the adult population of Belgium: report of a worksite study, Attention Hypertension. J Hum Hypertens 2002; 16: 47-52
3. Fagard et al. Treatment and blood pressure control in isolated systolic hypertension vs diastolic hypertension in primary care. Journal of Hypertension 2002; 20: 1297-1302
4. Erdine S. How well is hypertension controlled in Europe? ESH Newsletter 2011; 12:5-6
5. Redon et al. Why in 2016 are patients with hypertension not 100% controlled? A call to action. Journal of Hypertension 2016;34:1480-1488
6. Xie et al., A medication adherence and persistence comparison of hypertensive patients treated with single-, double- and triple-pill combination therapy Curr Med Res & Op. 2014;30:15-22.
7. Mancia G, Fagard R et al. 2013 ESH/ESC Guidelines for the management of arterial hypertension. Journal of Hypertension 2013, 31: 1281-1357.
8. Bogaert M et al. Guide d’évaluation des études non interventionnelles. http://www.fagg-afmps.be/fr/humain/medicaments/medicaments/recherche_developpement/comite_d_ethique/

***10.* *Signatures***

**Investigateur  Servier Benelux**

Nom : Nom : Van Nieuwenhuyse Bregt

Signature : Signature :


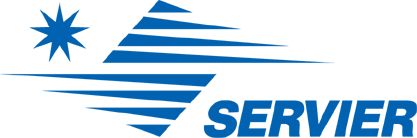

Supplement: S5 File — (DOC) [file pone.0248471.s006.doc]
