# Supplementary material for: Antihypertensive treatment in a general uncontrolled hypertensive population in Belgium and Luxembourg in primary care: Therapeutic inertia and treatment simplification. The SIMPLIFY study
Source: PLoS One. 2021 Apr 5;16(4):e0248471. doi: 10.1371/journal.pone.0248471 (PMC8021160; doi:10.1371/journal.pone.0248471)

**Observationele transversale studie om de motivatie en belangrijkste drijfveer van huisartsen om de bloeddruk verlagende behandeling in de algemene populatie van hypertensiepatiënten in België behandeld met tenminste 1 antihypertensivum te SIMPLIFIEREN of te intensifiëren na te gaan**

**Studieprotocol**

***Wetenschappelijke rationale***

Hypertensie is nog steeds een belangrijke doodsoorzaak en één van de voornaamste beïnvloedbare risicofactoren voor cardiovasculaire, cerebrovasculaire en renovasculaire aandoeningen (1).

Ondanks de beschikbaarheid van verschillende efficiënte geneesmiddelenklassen voor de behandeling van hypertensie (diuretica, bètablokkers, calciumantagonisten, ACE-remmers en angiotensinereceptorantagonisten), blijft de behandeling van vele hypertensiepatiënten suboptimaal. Volgens schattingen is bij de helft van de patiënten de hypertensie niet gekend, is bij ten minste de helft van de behandelde patiënten de bloeddruk niet onder controle en is de helft van de patiënten die behandeld worden met antihypertensiva op lange termijn niet therapietrouw (2-4). Deze suboptimale behandeling van hypertensie heeft een grote impact op de volksgezondheid en de kosten van de gezondheidszorg. Deze suboptimale behandeling van hypertensie patiënten is in grote mate gerelateerd aan een bestaande therapeutische inertie om een hoge bloeddruk adequaat onder controle te krijgen bij het medische korps (5). Artsen lijken omwille van verschillende factoren onvoldoende gemotiveerd om de behandeling van hun hypertensiepatiënten te optimaliseren.

Hypertensiepatiënten hebben veelal een hoge medicatielast en worden behandeld met meerdere tabletten per dag. Het is aangetoond dat de therapietrouw sterk afhankelijk is van het aantal genomen tabletten per dag. Patiënten behandeld met 3 verschillende tabletten per dag zijn 74% minder therapietrouw vergeleken met patiënten die behandeld worden met 1 tablet per dag (6). Om de therapietrouw te verbeteren wordt in de huidige aanbevelingen van de European Society of Hypertension en de European Society of Cardiology aangeraden vaste associaties van antihypertensiva te gebruiken (7). Bij gebruik van een bi- of tritherapie wordt in de Europese aanbevelingen de voorkeur gegeven aan één inname per dag, omdat beperking van het aantal innames per dag en van het aantal tabletten bevorderlijk is voor de therapietrouw en de bloeddrukcontrole. In deze aanbevelingen van de European Society of Hypertension en de European Society of Cardiology (7) wordt bovendien aangeraden om bij de behandeling van ongecontroleerde hypertensiepatiënten een molecule aan de behandeling toe te voegen eerder dan de dosis te verhogen. Dit omdat deze strategie inwerkt op verschillende werkingsmechanismen, waarvan verondersteld kan worden dat deze strategie efficiënter is dan een dosisverhoging.

In België zijn er weinig epidemiologische gegevens beschikbaar omtrent de mate van deze geldende therapeutische inertie en therapeutische beslissingsvorming in de behandeling van hypertensie. Met deze studie wordt nagegaan in welke mate de Belgische huisartsen gemotiveerd zijn om de behandeling van ongecontroleerde hypertensiepatiënten te intensifiëren of te vereenvoudigen. Bovendien wordt onderzocht welke factoren deze beslissingsvorming sturen om te identificeren op welke factoren de verschillende actoren in de gezondheidszorg kunnen inspelen om de suboptimale behandeling van hypertensiepatiënten te verbeteren.

***2. Doelstelling***

Het doel van deze studie is te evalueren in welke mate en op welke manier in de huidige praktijk van huisartsen in België de behandeling van ongecontroleerde hypertensie patiënten geïntensifieerd of vereenvoudigd wordt en dit in functie van:

- de bloeddruk (systolisch en diastolisch) en bloeddrukcontrole
- de door de arts geschatte therapietrouw aan de bloeddrukverlagende medicatie
- het aantal/type gebruikte antihypertensiva
- het totaal aantal gebruikte geneesmiddelen
- het aantal en/of type comorbiditeiten
- demografische factoren (leeftijd, geslacht, BMI,…)

*Verwachte toegevoegde waarde*

Door deze studie uit te voeren zullen we:

- de mate van therapeutische inertie voor de behandeling van hypertensie bij huisartsen in België kennen

- de factoren kunnen identificeren die huisartsen in België motiveren om de hypertensie behandeling te intensifiëren of te vereenvoudigen

- de therapietrouw in de populatie van niet gecontroleerde hypertensiepatiënten in België kunnen inschatten

***3. Opzet***

*Dit is een observationele transversale studie (cross-sectional survey)*.

Het studieontwerp heeft de volgende kenmerken:

- Enkel de bestaande gegevens die gewoonlijk worden geregistreerd in de dagelijkse praktijk, worden verzameld. Deze routinegegevens worden niet specifiek verzameld om het doel van de studie te bereiken.
- De gegevens worden op één bepaald moment verzameld, zodat we een transversaal overzicht krijgen van de bestudeerde populatie. De studie zal zich beperken tot de huidige situatie (in 2017).
- Het is een zuiver beschrijvende studie (de statische analyse kan de huidige situatie beschrijven). De ontwikkeling van deze situatie in de loop der tijd wordt niet onderzocht (dat is alleen mogelijk met een longitudinale studie).

De studie zal de gestelde vragen perfect kunnen beantwoorden.

Deze studie, die uitgaat van beschikbare gegevens en geen inbreng vraagt van de betreffende patiënten, wordt beschouwd als 'retrospectief' in de zin van paragraaf 1.4 van de Leidraad voor niet-interventionele studies van mei 2008 (8). De wet inzake experimenten op de menselijke persoon (7 mei 2004) is dus niet van toepassing. In deze context heeft het Visumbureau van pharma.be een voorafgaand visum verstrekt voor de studie, op basis van de aanbevelingen in de Deontologische code (23 maart 2012).

***4. Patiënten en onderzoeksartsen***

*4.1 Patiënten*

De patiënten waar de studie betrekking op heeft, zijn ongecontroleerde hypertensiepatiënten (≥ 18 jaar, BD ≥140/90 mm Hg) die reeds behandeld worden met minstens 1 antihypertensivum, en bij een huisarts op spreekuur komen in België. De patiënten met secundaire hypertensie worden uitgesloten uit de studie.

Het aantal patiënten, waar de gegevens van zullen verzameld worden, wordt geschat op 4.725.

Dit aantal is niet gebaseerd op een statistische berekening –wat inherent is aan het gebruikte type studieontwerp - maar komt overeen met de gebruikelijke steekproefgrootte voor dit soort epidemiologisch onderzoek.

*4.2 Onderzoeksartsen*

De studie richt zich op huisartsen die hun praktijk hebben in België en die dit type patiënten regelmatig zien op hun spreekuur.

Het aantal artsen dat noodzakelijk is om de gegevens van 4725 patiënten te verzamelen wordt geschat op 315, waarbij elke huisarts de gegevens verzamelt van 15 patiënten die in aanmerking komen voor de studie en die hij/zij op spreekuur ziet. Deze huisartsen worden gerekruteerd over heel België, met een evenwichtige geografische spreiding, in een periode van 3 maanden (16 Maart 2017 tot 8 Juni 2017). Op die manier kunnen de artsenbezoekers van Servier Benelux afspraken plannen met de onderzoeksartsen voor het eerste bezoek, hun toestemming voor deelname verkrijgen, het contract laten ondertekenen en het nodige materiaal voor de studie overhandigen.

Om selectiebias te voorkomen wordt aan de onderzoeksartsen gevraagd om de *laatste 15 opeenvolgende* patiënten op te nemen die voldoen aan de studiecriteria en die ze onlangs op spreekuur hebben gezien. De onderzoek artsen worden verondersteld toestemming tot deelname aan deze studie te vragen aan hun patiënten.

***5. Verzamelde gegevens***

Voor elke patiënt die in aanmerking komt, noteert de onderzoeksarts de volgende bestaande gegevens op een observatieformulier. De gegevens worden verzameld aan de hand van de medische dossiers van de patiënten. Er wordt niet gepland om tijdens toekomstige contacten (consultatie) met de patiënten observaties te doen:

- het nr. van de patiënt in de studie (van 1 tot 15),
- leeftijd, geslacht, lengte en gewicht
- het al dan niet voorkomen van een comorbiditeit (diabetes, cardiovasculaire antecedenten, stabiel vaatlijden, hartfalen, nierinsufficiëntie, aritmie, dyslipidemie, andere)
- systolische/diastolische bloeddruk (mmHg)
- door de arts geschatte therapietrouw aan de bloeddrukverlagende medicatie (goede therapietrouw, matige therapietrouw, niet therapietrouw)
- behandeling voor de consultatie: gebruikte antihypertensiva (merknaam, stofnaam en dosering), andere bijkomende medicatie, totaal aantal tabletten per dag
- medische beslissingsvorming tijdens de consultatie (aanpassen medicatieschema antihypertensiva: ja of nee)
- behandeling na de consultatie: gebruikte antihypertensiva (merknaam, stofnaam en dosering), andere bijkomende medicatie, totaal aantal tabletten per dag
- motivatie gebruik vaste associaties (indien van toepassing)

Naast de patiëntgegevens staan op het observatieformulier ook de naam van de onderzoeksarts, diens adres, handtekening en stempel, om zo nodig contact op te kunnen nemen en als garantie van authenticiteit.

Op het observatieformulier staan ook het visumnummer van pharma.be en de naam van de contactpersoon bij Servier Benelux, zodat de onderzoeksarts zo nodig aanvullende informatie kan opvragen.

De benodigde gegevens voor de studie worden verzameld in een periode van 4 maanden na de toestemming van de onderzoeksarts om deel te nemen aan de studie, zodat deze drukbezette huisartsen de tijd hebben om de 15 patiënten die in aanmerking komen voor de studie te bepalen en de bijbehorende observatieformulieren in te vullen. Daarna hebben de artsenbezoekers van Servier Benelux 2 maanden de tijd om de observatieformulieren in te zamelen. Aangezien de uiterste datum voor het rekruteren van onderzoeksartsen 8 Juni 2017 is, zouden alle verzamelde gegevens beschikbaar moeten zijn op 8 November 2017, de einddatum van de studie. De data verzameld in deze transversale studie is eigendom van Servier Benelux. Publicatie van gegevens uit de opgezette databank kan enkel na toestemming van Servier Benelux.

Als de onderzoeksarts gegevens voor de studie zoekt in het patiëntendossier en daarbij op een bijwerking stuit die mogelijk verband houdt met het gebruik van een geneesmiddel van Servier Benelux en die (nog) niet is gerapporteerd aan Servier Benelux, wordt hij/zij verzocht dat zo spoedig mogelijk te doen door het formulier voor Rapportering van bijwerking onmiddellijk te verzenden naar de verantwoordelijke voor pharmacovigilantie bij Servier Benelux, dr. Xavier Pottier (fax: 02/529.43.89 , e-mail: [pharmacovigilance@be.netgrs.com](mailto:pharmacovigilance@be.netgrs.com), tel: 02/529.43.11). Indien nodig zal de verantwoordelijke voor pharmacovigilantie van Servier Benelux contact opnemen met de onderzoeksarts om meer informatie in te winnen. Dit formulier voor rapportering van bijwerking is ook te gebruiken in volgende gevallen:

- Elk ongewenst effect
- Elke blootstelling aan een geneesmiddel tijdens de zwangerschap of borstvoeding
- Elke overdosering (opzettelijk of per ongeluk, misbruik of verkeerd gebruik
- Elk gebruik buiten indicatie (off-label)
- Elke professionele blootstelling
- Elk gebrek aan efficaciteit

***6. Analyse van de resultaten***

Na een kwaliteitscontrole worden de gegevens van alle correct ingevulde observatieformulieren ingevoerd in een Excel-tabel om de volgende beschrijvende statistische analyses te kunnen uitvoeren:

- studiepopulatie: aantal patiënten, verdeling op basis van demografische data (leeftijd, geslacht, BMI), cardiovasculair risico (comorbiditeiten, systolische en diastolische bloeddruk, door de arts ingeschatte therapietrouw aan de bloeddrukverlagende medicatie), en de behandeling (aantal gebruikte antihypertensiva, type antihypertensiva, totale medicatielast)
- systolische en diastolische bloeddrukcontrole: totale populatie versus risicopopulatie, associatie met door de arts ingeschatte therapietrouw aan de bloeddrukverlagende medicatie, relatie ten opzichte van totaal aantal gebruikte antihypertensiva, type antihypertensiva, en totale medicatielast
- medische beslissingsvorming: relatie intensificatie of vereenvoudiging antihypertensieve behandeling ten opzichte van demografie, risicofactoren, systolische en diastolische bloeddruk, door de arts ingeschatte therapietrouw aan de bloeddrukverlagende medicatie, totaal aantal gebruikte antihypertensiva, type gebruikte antihypertensiva, en totale medicatielast.
- Motivatie gebruik van vaste associaties in de behandeling van hypertensie

***7. Studieverslag en publicatie***

Na analyse van de gegevens en interpretatie van de resultaten wordt een studieverslag opgesteld en goedgekeurd door de wetenschappelijke dienst van Servier Benelux. Er is ook een publicatie in een wetenschappelijk tijdschrift gepland.

Dit verslag (of deze publicatie) wordt verzonden naar alle onderzoeksartsen die hebben deelgenomen aan de studie en wordt ook ter beschikking gesteld van de organen van pharma.be vermeld in artikel 52, § 1 van de Deontologische code van 23 maart 2012.

***8. Kwaliteitsbeheer***

Het studieprotocol is opgesteld in samenwerking met de wetenschappelijke dienst van Servier Benelux, die het heeft goedgekeurd en die toeziet op het goede verloop van de studie.

Het Visumbureau van pharma.be heeft een voorafgaand visum verstrekt voor de studie, op basis van de aanbevelingen in de Deontologische code (28 maart 2014).

Om de kwaliteit van de verzamelde gegevens te controleren vindt er een kwaliteitscontrole plaats, waarbij rekening wordt gehouden met de kwaliteitsrisico's die inherent zijn aan de methodologie van deze observationele studie. Daarvoor zal de wetenschappelijke dienst van Servier Benelux bij wijze van steekproef 5% van de observatieformulieren van de onderzoeksartsen die hebben deelgenomen aan de studie, verifiëren.

De wetenschappelijke dienst van Servier Benelux beheert de gegevens, voert de statistische analyses uit en stelt het studieverslag op.

De rol van de artsenbezoekers van Servier Benelux blijft beperkt tot de twee volgende acties:

- eerste bezoek: voorstelling van de studie, overhandiging van het protocol en observatieformulier, ondertekening van het contract met de onderzoeksarts
- afsluitend bezoek: ophaling van de ingevulde observatieformulieren

***9. Referenties***

1. Lewington S et al. Age-specific relevance of ususal blood pressure to vascular mortality: a meta-analysis of individual data for one million adults in 61 prospective studies. Lancet 2002; 360: 903-913
2. Duprez et al. Prevalence of hypertension in the adult population of Belgium: report of a worksite study, Attention Hypertension. J Hum Hypertens 2002; 16: 47-52
3. Fagard et al. Treatment and blood pressure control in isolated systolic hypertension vs diastolic hypertension in primary care. Journal of Hypertension 2002; 20: 1297-1302
4. Erdine S. How well is hypertension controlled in Europe? ESH Newsletter 2011; 12:5-6
5. Redon et al. Why in 2016 are patients with hypertension not 100% controlled? A call to action. Journal of Hypertension 2016;34:1480-1488
6. Xie et al., A medication adherence and persistence comparison of hypertensive patients treated with single-, double- and triple-pill combination therapy Curr Med Res & Op. 2014;30:15-22
7. Mancia G, Fagard R et al. 2013 ESH/ESC Guidelines for the management of arterial hypertension. Journal of Hypertension 2013, 31: 1281-1357.
8. Bogaert M et al. Guide d’évaluation des études non interventionnelles. http://www.fagg-afmps.be/fr/humain/medicaments/medicaments/recherche_developpement/comite_d_ethique/

***10. Handtekeningen***

**Onderzoeksarts Servier Benelux**

**Naam:** Naam: Van Nieuwenhuyse Bregt

Handtekening: Handtekening:


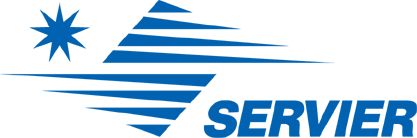

Supplement: S6 File — (DOC) [file pone.0248471.s007.doc]
